# Impact of population tobacco control interventions on socioeconomic inequalities in smoking: a systematic review and appraisal of future research directions

**DOI:** 10.1136/tobaccocontrol-2020-055874

**Published:** 2020-09-29

**Authors:** Caroline E Smith, Sarah E Hill, Amanda Amos

**Affiliations:** 1 Usher Institute, University of Edinburgh, Edinburgh, UK; 2 Global Health Policy Unit, University of Edinburgh, Edinburgh, UK

**Keywords:** disparities, socioeconomic status, public policy

## Abstract

**Background:**

While price increases and targeted cessation support have been found to reduce inequalities in smoking by socioeconomic status (SES), evidence on other measures is mixed. We aimed to update the most recent (2014) previous review by identifying and appraising evidence published since 2013 on the equity impact of population tobacco control measures.

**Methods:**

Systematic searching of 10 electronic databases and hand-searching of four key journals identified 68 primary research articles published since 2013 that sought to examine the equity impact of population tobacco control measures in high-income countries with a negative socioeconomic gradient in smoking. Reported equity impacts were categorised as positive (greater impact among lower SES), neutral (no difference by SES), negative (greater impact among higher SES) or mixed/unclear.

**Results:**

There was substantial growth in research seeking to evaluate the equity impact of tobacco control interventions, but the majority of new studies showed mixed/unclear results. Findings for price increases and targeted cessation support continue to suggest an equity-positive impact, but limitations in the available evidence make further assessment difficult. Substantial differences in the context, scale and implementation of tobacco control policies make straightforward comparison of findings from the previous 2014 and current reviews problematic.

**Conclusion:**

Researchers need to adopt more sophisticated, multidisciplinary approaches in evaluating the equity impact of tobacco control measures—developing robust measures of equity effect and using frameworks that take account of context, existing systems/processes and the likely mechanisms of action. Socioeconomic differences in intervention impact within low-income and middle-income countries require evaluation.

## Introduction

The development of effective tobacco control measures^1^—supported by the WHO Framework Convention on Tobacco Control^2^—has been highly successful in reducing both tobacco consumption and the burden of tobacco-related diseases in many countries,^3^ with the greatest gains experienced by high-income countries with strong regulatory frameworks.^4^ Yet many high-income countries have seen the emergence of significant social inequalities in tobacco use as overall smoking has declined,^5–7^ with evidence persistently showing higher smoking levels among groups with lower socioeconomic status (SES)^5–7^—namely, those positioned lower in the social hierarchy who experience reduced access to society’s economic, social and political resources.^8 9^ Recent WHO data for Western Europe indicate smoking rates are typically two to three times greater among the most disadvantaged,^10^ posing a challenge for both health equity and aspirations of a tobacco endgame.

Several systematic reviews^11–13^ have sought to summarise evidence on the impact of tobacco control interventions on socioeconomic inequalities in smoking (equity impact); the most recent by Brown *et al* included evidence published up to February 2013.^12^ While a range of population interventions are effective at reducing smoking prevalence overall,^3^ price/taxation measures are the only intervention to demonstrate consistent evidence of an equity-positive impact (namely, tobacco price increases lead to greater smoking declines among low SES groups).^11–13^ Equity impacts of other tobacco control interventions are less clear, although there is some evidence that national stop smoking services can achieve equity-positive effects on quitting by reaching proportionally more low SES smokers to compensate for their lower quit rates.^12^

Given the evolution of both tobacco control policies and related research over recent years, it is timely to update these reviews by examining evidence published since 2013.^12^ We therefore aimed to identify and appraise peer-reviewed primary research evidence assessing the equity impact of population-level interventions in high-income countries where smoking inequalities are an issue. This updated review has three main objectives:

1. Summarise current evidence on the equity impact of tobacco control measures on socioeconomic inequalities in smoking and compare findings with the most recent previous review^12^;
2. Explore the challenges of assessing changes in intervention equity impact within a complex and evolving policy context;
3. Illustrate these challenges via a case study and detailed synthesis of the evidence for one exemplar intervention, namely, smoke-free policies.

## Methods

This systematic review draws on the conceptual framework used by Brown *et al* 12 where population tobacco control interventions are defined^14^ as “those applied to populations, groups, areas, jurisdictions or institutions with the aim of changing the social, physical, economic or legislative environments to make them less conducive to smoking” and categorised into five main intervention types (table 1). The protocol was prospectively registered on PROSPERO (https://www.crd.york.ac.uk/PROSPERO/display_record.php?RecordID=96686) and the review written in accordance with the Preferred Reporting Items for Systematic Reviews and Meta-Analyses (PRISMA): Equity Reporting Guidelines.^15^

**Table 1:** Systematic search and data extraction specification

Main intervention types	Price and taxation measures; smoke-free policies; mass media campaigns; sales and marketing controls; population-level cessation support
Smoking-related outcome measures	Smoking prevalence and consumption; quit behaviours and success; intermediate outcomes (eg, awareness and attitudes); secondhand smoke exposure and smoke-free coverage; retailer density and compliance
SES indicators	Individual measures (eg, education, income and occupational group); area-based measures (eg, neighbourhood affluence or deprivation, poverty, median household income)
Bibliographic databases	ASSIA; BIOSIS; CINAHL Plus; Cochrane Library; EMBASE; IBSS; Medline; PsycINFO; Sociological Abstracts; Web of Science Core Collection
Online journals (for hand-search)	*Addiction*; *Nicotine & Tobacco Research*; *Social Science & Medicine*; *Tobacco Control*
Data extraction items	Intervention details; research design; geographical location; data collection period; sample characteristics; smoking-related outcomes; SES indicators

SES, socioeconomic status.

### Eligibility criteria

Studies were eligible for inclusion if they evaluated a population tobacco control intervention, comparing a smoking-related outcome across two or more SES groups, with relevant outcomes reflecting the full spectrum of potential mechanisms via which tobacco use might be reduced (table 1). Eligible studies were published in English from the beginning of 2013 (but not included in the previous review) and focused primarily on non-hospitalised adults over 18 (although several studies had an age limit of 15/16). We restricted our review to countries at stage 4 of the tobacco epidemic^16^ and with a negative socioeconomic gradient in smoking^17^ (ie, high-income countries with existing inequalities). Population interventions involving e-cigarettes were not considered as they were covered by a recent review of the equity impact of non-combustible nicotine products.^18^

### Search strategy and study selection

Studies were identified through electronic searches of 10 bibliographic databases (table 1) on 10 May 2018, supplemented by hand-searching of four key journals to identify pending publications. Search terms covered smoking, socioeconomic inequalities and population tobacco control interventions, but no restrictions were placed on research design (table 1 and online supplemental appendix S1). Study selection involved a multi-step process, beginning with preliminary screening where two reviewers (CES and an RA) each scanned half the abstracts to identify those evaluating a population tobacco control intervention within a stage 4 country which included a reference to analyses by SES. Borderline cases were cross-checked by the other reviewer. Selected papers were then subject to more detailed abstract, and finally full-text, reviews against the complete set of eligibility criteria, with all decisions being independently verified by at least one other author. Differences of opinion were resolved through discussion.

10.1136/tobaccocontrol-2020-055874.supp1 Supplementary data

### Data extraction, quality assessment and data synthesis

Standardised data extraction sheets were used to record basic design details for each eligible study (table 1 and online supplemental appendix S2), with findings by SES being summarised separately (online supplemental appendix S3). Quality was assessed using a shortened checklist derived from the Critical Appraisal Skills Programme (CASP) tools^19^ which focused on four key items of most relevance across all research designs, with studies being given a ‘value to review’ score between 0 and 6. Full systematic quality appraisal results are in online supplemental appendix S4. Additional reflections on other quality issues applying only to individual, or a subset of, studies are in online supplemental appendix S3. All data extraction and quality appraisal was initially undertaken by CES and checked by another author.

We present our findings via narrative synthesis in three parts, mirroring our review aims. First, we provide an updated assessment of equity impact by intervention type, using the rating scale developed by Brown *et al* 12 (table 2) and comparing overall findings with those from the 2014 review. We then reflect on the limitations of this approach in exploring changes in intervention equity impact, highlighting how a simple rating scale cannot adequately capture the full complexity of evidence which is evolving in diverse but nuanced ways. Finally, we provide a detailed account of the evidence relating to one exemplar intervention to illustrate the challenges in understanding how equity impact evolves across disparate intervention formulations, contexts and populations. Smoke-free was retrospectively chosen as the exemplar since it best reflects the range of issues faced.

**Table 2:** Equity impact classification criteria

Equity impact	Classification criteria
Positive	Evidence that lower SES groups are relatively more responsive to intervention
Neutral	No evidence that intervention has differential impact across low and high SES groups
Negative	Evidence that higher SES groups are relatively more responsive to intervention
Mixed/unclear	Mixed equity effects and/or unable to assess overall intervention equity impact

Source: Brown *et al* (2014) Equity impact of population-level interventions and policies to reduce smoking in adults: a systematic review. *Drug and Alcohol Dependence*.

SES, socioeconomic status.

## Results

### Overview of included studies

**Figure 1:**
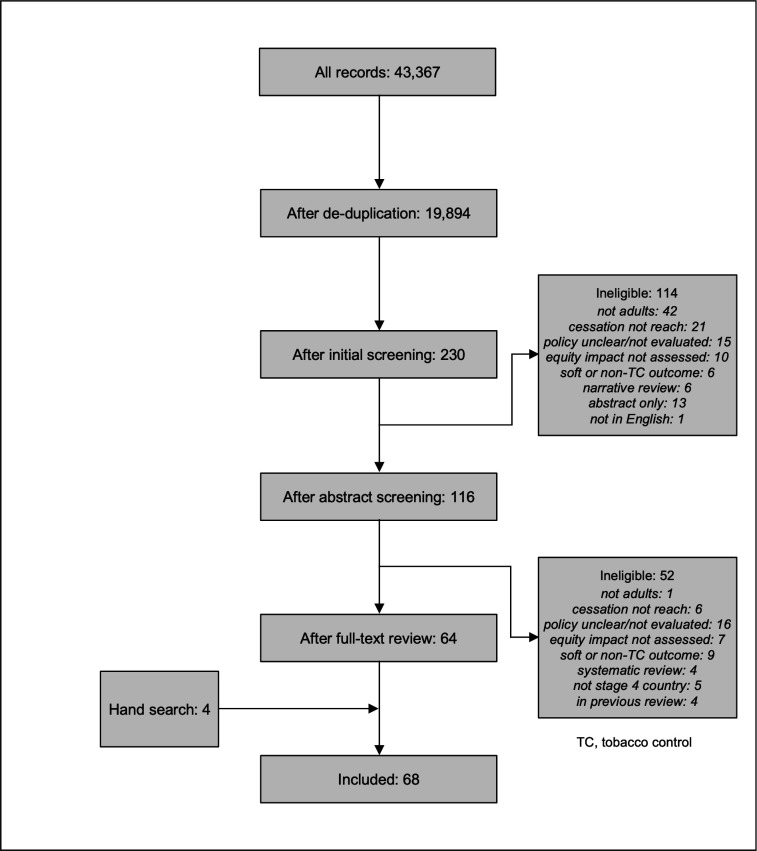
PRISMA flow diagram.

Bibliographic database searches identified 43 367 papers which reduced to 19 894 once duplicates were removed (figure 1). Of these, 19 664 were excluded following initial title/abstract screening. A further 114 were judged ineligible through the detailed abstract review and 52 through the full-text review, with most excluded because they focused on young people not adults (43), because the policy was unclear or not evaluated (31), or because the study examined cessation support but did not assess population reach (27). The remaining 64 references were combined with 4 identified through hand-searching to give 68 papers for inclusion. Six studies evaluated multiple intervention types, with separate results being reported for a total of 82 interventions.

**Figure 2:**
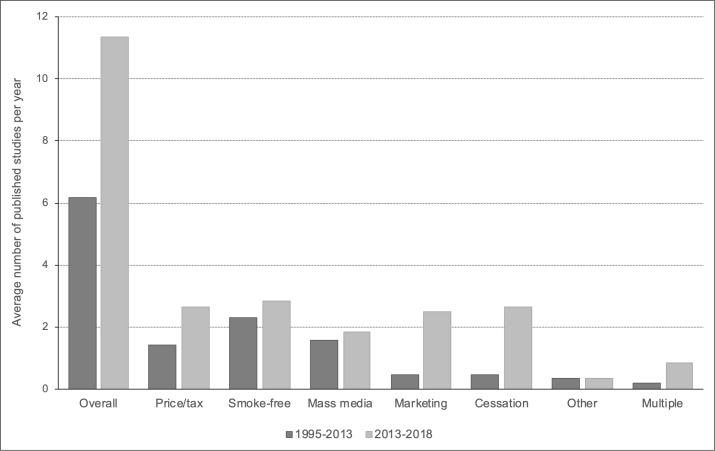
Annual publication rate by intervention type and review period.

Studies were split fairly evenly across the five main intervention types, with 16 evaluating the equity impact of tobacco price/taxation increases,^20–35^ 17 focusing on smoke-free policies,^20 27 32 35–48^ 11 on mass media campaigns,^20 24 35 49–56^ 15 on sales and marketing controls,^20 27 57–69^ and 16 investigating the impact of population-level cessation support.^20 27 70–83^ Two studies looked at broader inequalities-focused interventions^84 85^ and five evaluated the combined impact of multiple policies.^20 24 26 86 87^ Compared with the previous review, there was a substantial increase in the annual publication rate which nearly doubled from 6.2 papers per year during 1995–2013 (the period covered by Brown *et al* 12) to 11.3 during 2013–2018 (figure 2). Increases in the publication rate were seen across most intervention types, but were particularly evident for controls on tobacco sales and marketing, and for population-level cessation support.

The quality of the available evidence was mixed, with a mean ‘value to review’ score of 4.0 out of 6. Only half the included studies (35 out of 68) were designed with a primary focus on assessing intervention impact by SES; consequently, many studies were under-powered to detect differences by SES (only 24 of 82 intervention-specific analyses were adequately powered for all comparisons), and in some cases the study design and/or analysis was not optimised for assessing impact by SES. A reliance on retrospective, self-report data drawn from existing population surveys cast doubt on the validity of some outcome measures (although it did provide access to large representative samples). The common use of observational rather than experimental or quasi-experimental designs made it difficult to attribute observed changes to the intervention. Study quality was broadly similar across intervention types (mean score: price/taxation=4.2; smoke-free=3.8; mass media=3.9; marketing controls=3.5; cessation support=4.4). While direct comparison of quality scores was not possible across the two reviews (the current review used an updated and consolidated quality checklist which draws on the latest version of the CASP appraisal tools^19^ and uses a single checklist for all study types), there was no evidence of a general shift in quality: papers appeared in similar journals and the same issues of suboptimal study design and lack of attributability were encountered.

### Equity impact ratings: updated summary and comparison with 2014 (objective 1)

In terms of intervention equity impact, similar numbers of studies indicated an equity-positive (17) and an equity-negative (16) effect, broadly matching the pattern seen in the previous review (table 3 and online supplemental appendix S3). Only one equity-neutral result was reported this time, whereas Brown *et al* 12 found 36. The proportion of studies showing mixed/unclear findings increased dramatically from 18% (23) to 59% (48), with the majority (36 of 48) reporting complex equity effects across different outcome measures, SES indicators and/or demographic groups—a development that the summary equity impact ratings were not adequately able to capture. Mean quality scores did not differ markedly by equity impact (positive=4.5; negative=4.2; mixed/unclear=3.8).

**Table 3:** Overall equity impact results by intervention type and review

	Brown review^12^					Current review				
	Positive	Neutral	Negative	Mixed	Total	Positive	Neutral	Negative	Mixed	Total
Price/taxation	14	6	4	3	27	7	0	1	8	16
Smoke-free	3	10	25	6	44	2	0	7	8	17
Mass media	8	5	8	9	30	1	0	1	9	11
Marketing controls	2	7	0	0	9	1	1	4	9	15
Cessation support	4	2	0	3	9	6	0	2	8	16
Other	2	4	1	0	7	0	0	1	1	2
Multiple	0	2	0	2	4	0	0	0	5	5
All interventions	33	36	38	23	130	17	1	16	48	82

Equity impacts varied by intervention type, but were mostly in the same direction as those reported in the 2014 review. Among studies showing a clear equity result, findings for price/taxation measures and population-level cessation support continued to suggest an overall equity-positive effect, with greater impact among lower SES groups. Equity-negative findings continued to dominate studies examining smoke-free policies, while evidence around mass media campaigns remained mixed. Our review did though find a shift in evidence on sales and marketing controls, with findings weighted towards an equity-negative impact—compared with a predominantly neutral effect in the previous review. Looking beyond the overall summary measures, it appears that this shift may be partly explained by a change in the specific types of control evaluated.

### Equity impact ratings: what they don’t tell us (objective 2)

Our summary findings point to two key issues concerning the use of overall equity impact scores—such measures are no longer robust in the face of increasingly complex patterns of equity effect and they obscure the evolution and innovation that has occurred since the last review. These issues are further compounded by substantial diversity, complexity and change within the evidence base, and particularly in relation to intervention context, scale and implementation (ie, the specific intervention variant), which makes straightforward interpretation and comparison of summary measures problematic. We now explore this complexity in more detail.

#### Context

As in 2014, studies were heterogeneous in the populations covered. There was a predominance of US papers, with half (35 of 68) being carried out in America. Most of the rest (24) focused on Europe, looking primarily at the UK (10), Spain (3), the Netherlands (3) or multiple European countries (5). Six studies were undertaken in Australia, and two in Canada, with the final paper covering Australia and Canada. Most studies used individuals as the unit of analysis, drawing variously on members of the general population as well as more specific groups such as smokers and ex-smokers, pregnant women, employees and multi-unit housing residents/owners. Other units of analysis included households, organisations and geographical areas. Study sample sizes ranged from 4 to 17.7 million (most between 500 and 500 000). Data collection intervals were varied, with some studies gathering data over several weeks but others using multiple waves of data collection over many years (the longest being 24 years)—and, while a number of studies included data from as far back as 1984, most data were gathered between 2000 and 2017.

#### Scale

Across both reviews, approximately three-quarters of studies focused on policies and interventions delivered at national or state level although evaluation was not always undertaken at the same geographical level as delivery. For example, Choi and Boyle^21^ explored the impact of US federal tobacco tax increases on quitting among smokers in Minnesota whereas several studies assessed the equity impact of state-level cigarette taxes/prices across the USA as a whole.^23 25 28 29 33–35^ In other cases, the scale of delivery was more ambiguous. An Australian study of exposure to anti-tobacco TV advertisements in Victoria by Durkin *et al* 49 looked at the combined effect of state and federally funded campaigns. Hummel *et al*,^27^ moreover, relied on self-report data about whether four different tobacco control policies had led to thoughts of quitting in six European countries but provided no details regarding the policy jurisdictions. Alongside these national/state-level interventions, a significant minority of studies focused on more locally delivered policies (ie, county, city or community) or on voluntary interventions applied by organisations. Here, there were some changes across the two reviews. While around a quarter (7 of 30) of mass media studies in the 2014 review evaluated local-level campaigns, only one such study was identified in 2019.^55^ The reverse pattern was seen in relation to sales and marketing controls, where three studies^57 60 68^ of local/voluntary sales bans were found for 2013–2018 compared with none previously. In relation to voluntary smoke-free policies, the emphasis shifted from the workplace to multi-unit housing, with three studies assessing owner restrictions on smoking in such accommodation.^37 39 40^

#### Implementation

For nearly all intervention types, we found a shift in the specific variants of the tobacco control measures studied. Evaluations of smoke-free policies (see objective 3 below for more detail) broadened from a sole focus on restrictions covering indoor public spaces and workplaces to include measures covering outdoor public^41 45^ and private places.^37 39 40 44^ Where previously studies of mass media campaigns were split between those looking to boost quit intentions/attempts and those looking to increase uptake of cessation support, in the current review studies also began to examine the adoption of smoke-free homes.^35 51^ Comparisons of different forms of campaign content became more common, with Durkin *et al* 49 and Nonnemaker *et al* 54 exploring the role of emotion, Kim *et al* 50 looking at the use of stigmatising (vs non-stigmatising) messages, and Rayens *et al* 55 comparing the equity impact of loss-framed and gain-framed content. The majority of mass media studies across both reviews focused on traditional media platforms such as TV and radio. Only one study in each review explicitly mentioned using an online advertising campaign, with around a third of studies also reporting on the evaluation of multimedia campaigns. While studies of sales and marketing controls continued to concentrate primarily on cigarette packaging, there was a move from focusing on warnings labels to evaluating pack inserts and/or standardised packs (often alongside the introduction of pictorial warning labels).^63 65–67 69^ For the first time, three studies reported on the equity impact of retailer bans, with one covering menthol cigarettes sales near schools^57^ and two covering sales of tobacco products by pharmacies.^60 68^ The largest shift in intervention type related to population-level cessation support. Where in 2014, two-thirds of studies looked at provision of combined behavioural and pharmacological support (primarily by specialist services), here the focus was on brief interventions within primary care^73–77 82^ or access to free or low-cost cessation medication.^27 70 78 81^

### Equity impact ratings: the case of smoke-free policies (objective 3)

To further illustrate the diversity, complexity and change inherent to the evidence base, we next provide a detailed account of studies assessing smoke-free policies. We saw earlier that the weight of evidence across both reviews pointed to an equity-negative effect, with around half the studies finding a greater impact in high compared with low SES groups. More specifically in the current review, seven studies suggested an equity-negative impact,^20 36 37 39 41 43 46^ only two reported a positive effect^27 35^ and the remaining eight were mixed/unclear.^32 38 40 42 44 45 47 48^ While this overall similarity in results might indicate that changes have been limited, there have in fact been several key shifts in the evidence.

First, the 2014 review almost exclusively focused on smoking restrictions covering workplaces and/or indoor public spaces, demonstrating a clear distinction between national comprehensive policies that were largely equity positive or neutral, and voluntary, partial and regional bans that were predominantly equity negative. In the latest review, only three studies evaluated national comprehensive policies,^32 43 48^ with results suggesting a more mixed and nuanced equity picture. Two studies assessed the impact of smoke-free legislation covering public places in Spain. Regidor *et al* 32 found evidence of an initial equity-positive effect on the population quit ratio followed by a longer-term drift towards equity-negative impacts (among males). Lidón-Moyano *et al* 43 reported a possible equity-negative effect of the same legislation on the voluntary adoption of smoke-free homes. Evaluation of similar smoke-free legislation in Luxembourg^48^ found inconsistent and inconclusive results for smoking prevalence but clearer evidence of an equity-positive effect for quitting as a result of the ban.

In addition, Huang *et al* 42 assessed the coverage of local comprehensive smoke-free laws (encompassing workplaces, restaurants and bars) in 10 US states not already subject to state-level legislation. Here, results pointed to an equity-negative effect in most areas but an equity-positive effect in several states with more extensive smoke-free coverage, leading the authors to suggest that equity impact may evolve as smoke-free policies become more established. Two further papers^20 35^ explored the association between the strength of smoke-free legislation (measured by composite smoke-free law scores) and a range of outcomes measures, again demonstrating inconsistent equity effects. While Bosdreisz *et al* 20 found that higher ratings on the (domain-specific) Tobacco Control Scale were associated with wider inequalities in consumption and quitting across 27 EU countries, Zhang *et al* 35 reported narrowing inequalities in home smoking bans with strengthening state-level legislation in the USA.

Four papers in the current review focused on partial smoke-free regulations that applied to a more restricted range of locations, with one evaluating regional legislation covering hospitality venues in Geneva (Switzerland),^46^ two examining state-level restrictions (relating to workplaces *or* hospitality venues) in the USA^38 47^ and one looking at workplace smoking bans (resulting from state-wide, local-level or voluntary policies) among American employees.^36^ While none of these studies reported an equity-positive effect (two were negative^36 46^ and two mixed/unclear^38 47^), Babb *et al* 36 noted that occupational inequalities in workplace coverage in the USA narrowed between 2003 and 2010–2011 as such policies became more widespread. Furthermore, a survey-based analysis of policy triggers for thinking about quitting across six European countries found that smoking restrictions in public places largely had an equity-positive impact although the nature of these restrictions (ie, whether they were comprehensive or partial) was not recorded.^27^

Alongside this increasingly complex picture around workspaces and indoor public places, other changes in the evidence also began to emerge, reflecting the evolution of smoke-free regulation and its expansion into new spaces, such as outdoor public places and private areas. Two US papers explored smoke-free park policies, both focusing on partial bans that applied to some rather than all of the parks in a jurisdiction, with one national study showing an equity-negative effect on coverage at the county level^41^ and one a mixed/unclear effect on consumption across four parks within a single city.^45^ Murphy-Hoefer *et al* 44 evaluated a state-wide ban (Maine, USA) on smoking in vehicles carrying children, finding a possible equity-positive effect on the voluntary adoption of a complete smoke-free car rule but a mixed/unclear effect on the adoption of a similar smoke-free home rule. Several papers (all US-based) looked at smoke-free building policies instituted by owners of multi-unit housing blocks. Here, two studies reported an equity-negative effect on coverage^37 39^ while another found an equity-positive effect for coverage but an equity-negative effect for smoke incursions into individual housing units.^40^

## Discussion

Our review finds substantial growth and increasing complexity in the evidence assessing the impact of population tobacco control interventions on socioeconomic inequalities in adult smoking. Average annual publications almost doubled between 1995–2013 and 2013–2018, suggesting greater research interest in this understudied area,^12^ although this increased interest did not afford an improved understanding of equity impact. Findings for price/taxation measures and targeted cessation support continue to suggest an equity-positive impact, but otherwise the high proportion of studies with mixed/unclear equity impacts (increasing from 18% to 59%), together with the changing composition of the evidence base, makes it difficult to justify a direct comparison of the two reviews. Thus, despite a substantially expanded evidence base, we cannot provide a clearer indication of the equity impact of population tobacco control interventions than that previously offered.^11–13^

We encountered several challenges in assessing changes in intervention equity impact. First, reports of complex equity effects—where the direction of equity impact varied across different population groups, SES indicators and/or outcomes—were increasingly common. The reasons for this were not immediately apparent (the use of multiple SES indicators and outcome measures within a single study was, for example, similar across both reviews) but could suggest less substantive developments in some intervention areas (eg, in relation to price/taxation) and/or an increased willingness to publish studies where equity findings are less clear cut.

Both reviews were characterised by considerable heterogeneity in intervention context and scale, with studies focusing on different units of analysis (from individuals to organisations and geographical areas), different periods of intervention and evaluation (with the current review alone including data back to 1984), and different jurisdictions (from national legislation to local neighbourhood interventions and voluntary institutional policies). Given this high degree of heterogeneity, it is far from certain that the two reviews are comparing like with like—a problem further compounded by high levels of intervention innovation, with shifts in the specific formulation of policies/measures employed across most intervention types. The WHO, moreover, recognises that measures such as smoke-free policies, mass media campaigns and marketing controls can be introduced with different degrees of comprehensiveness,^88^ yet most studies we reviewed did not attempt to categorise the strength of the intervention evaluated, further limiting our ability to judge comparability.

Taken together, our findings highlight the difficulties of trying to synthesise findings from evaluations of complex policy approaches where the available tools for evaluating and systematically reviewing intervention impacts are largely derived from individual-level interventions. We argue that the complex mechanisms via which population tobacco control interventions have their effects—combined with diversity in their implementation, scale and effects in different population groups—require the development of more nuanced approaches to evaluating their impacts on socioeconomic inequalities in smoking. Encouragingly, we saw initial evidence of attempts to reflect these subtleties in the analysis and interpretation of equity effects, with researchers beginning to consider how SES might interact with other aspects of social location and context, to discuss how different tobacco control measures might interact with one another, and to explore how the impact of particular policies may change over time. Thus—for example—Huang and colleagues^42^ noted that the effect of local smoke-free policies may be influenced by both state-level tobacco control programmes and the specific characteristics of the local population. Two studies exploring the equity impact of bans on pharmacies selling tobacco products (one state level^68^ and one city level^60^) found that equity effects were dependent on the socially patterned distribution of pharmacies. Zhang *et al*,^35^ in contrast, evaluated the impact of three different interventions (tobacco tax, smoke-free laws and media campaigns) on home smoking bans, assessing their effects both separately and jointly, and finding that the equity-positive effect of smoke-free laws was no longer apparent in the multi-policy analysis. The study by Huang *et al*,^42^ moreover, suggested that the equity impact of local smoke-free laws is partly dependent on how long these laws have been in place and thus on how far non-smoking norms have become established.

### Limitations of review

While we sought to update the most recent previous review,^12^ we made some minor changes in our eligibility criteria to reflect changes in the available evidence base (an overall increase in volume combined with the use of innovative interventions and evaluative approaches). We slightly tightened our eligibility criteria by further limiting the current review to countries with a negative socioeconomic gradient in smoking^17^ and by restricting our definition of population tobacco control interventions to those applied to non-hospitalised populations. As a result, we did not strictly replicate the search and inclusion strategy used in Brown *et al*.^12^ Our focus on understanding intervention impact on existing socioeconomic inequalities in smoking—rather than on the broader issue of differential intervention impact by SES—also means that our findings may not be applicable beyond high-income countries.

While we appraised the quality of individual studies identified in this review, we did not consider it relevant or appropriate to set a minimum quality threshold for inclusion since our primary finding relates to the increasing complexity and diversity of the evidence base around intervention equity impact. Our review therefore includes studies of lower quality and/or of modest value to our aim.

Our initial data synthesis summarises the evidence base by counting reported study outcomes by categories of ‘equity impact’, giving numbers (overall and by intervention type) indicative of ‘positive’, ‘neutral’, ‘negative’ or ‘mixed/unclear’ equity impacts. While these numbers provide a crude summary of the available research in terms of equity effects, we emphasise that these figures should not be interpreted as a robust evaluation of the balance of evidence since (1) a single study might contribute more than one finding (where more than one measure of SES or outcome is used); (2) these figures do not account for study quality; and (3) the results of individual studies are not directly comparable, given differences in indicators of exposure (eg, individual vs area-level SES) and outcome (eg, smoking prevalence, intervention awareness, quit attempts, retailer density), differences in assessment of intervention impact by SES (eg, relative reduction in cigarette consumption, absolute difference in retail outlet density) and differences in study context.

### Implications for research

While our review reflects increased research interest in impacts of tobacco control on socioeconomic inequalities in smoking, there are several areas where the value of such research could be enhanced. The quality of the available evidence is limited by weaknesses in study design and power, often reflecting a lack of focus on equity impacts in the initial study aim. There is a clear need for more studies evaluating the equity impact of tobacco control interventions to be specifically designed and powered to be able to detect any shift in inequalities. Stronger study designs include those that collected data before and after the introduction of an intervention and/or those with a control group, and those that used study instruments (such as surveys) specifically designed to assess the equity impact of the relevant intervention. Alongside this, research is also required on differential intervention impacts by SES in low-income and middle-income countries where smoking inequalities are still evolving.

A key implication of our review is the need for researchers to develop more sophisticated and multidisciplinary approaches to evaluating the impact of tobacco control measures, particularly with reference to their potential impact on socioeconomic inequalities in smoking. Several researchers have recognised the need for a broader range of tools in evaluating complex health interventions, with suggested approaches including process evaluation,^89^ systems perspectives^90 91^ and realist evaluation.^92^ Common elements of these approaches include attention to context, recognition that the impact of interventions depends on interactions with existing systems and processes, and use of theory as a framework for interpreting and synthesising empirical evidence. Systems perspectives and realist evaluation approaches are particularly relevant in considering how to synthesise findings from a range of studies, with the latter having been used successfully to consider the differential effects of tobacco control interventions by socioeconomic position among adolescents.^93–96^

We argue that health researchers need to make greater use of these approaches in exploring the impact of tobacco control interventions by SES. This almost certainly precludes further attempts (such as ours) to synthesise evidence across diverse approaches and contexts. Instead, we recommend reviews focusing on specific interventions that can take account of evidence from a range of methods and disciplines, applying this to an underlying theory of the relevant mechanisms via which the intervention is thought to have its effect. Such an approach requires a narrower but deeper evaluation of relevant evidence, in contrast with the more comprehensive approach employed in most systematic reviews. This may involve some adjustment in the expectations of readers, reviewers, editors and research funders who may be more familiar with the empiricist assumptions underlying most systematic reviews of health interventions. However, the advantage of these more theoretically informed and multidisciplinary approaches is a more nuanced understanding of how tobacco control interventions interact with existing regulations and social structures, and thus a better understanding of how they might be adjusted or tailored to meet the needs of less advantaged groups. Ultimately, this understanding is necessary if we are to reduce socioeconomic inequalities in smoking and make genuine progress towards a tobacco endgame.

**What this paper adds**

- Previous systematic reviews suggest that price increases and targeted population-level cessation support are the interventions most likely to decrease socioeconomic inequalities in smoking, while other interventions have mixed effects.
- We updated the most recent systematic review (from 2014) on the impacts of population tobacco control measures on inequalities in smoking by socioeconomic status (SES).
- While a growing number of studies have sought to evaluate the equity impacts of tobacco control interventions, the majority of new studies show mixed or unclear results. Price increases and targeted population-level cessation support continue to be the only interventions where there is consistent evidence of a greater effect among low-SES smokers.
- In order to assess the equity impacts of tobacco control interventions in the real world, researchers need to develop more sophisticated and multidisciplinary evaluative approaches (including robust measures of equity effect) which take account of variations in context, the interaction of specific tobacco control interventions with existing systems and processes, and the mechanisms via which interventions produce their effects.
