# Supplementary material for: Impact of population tobacco control interventions on socioeconomic inequalities in smoking: a systematic review and appraisal of future research directions
Source: Tob Control. 2020 Sep 29;30(e2):e87–95. doi: 10.1136/tobaccocontrol-2020-055874 (PMC8666809; doi:10.1136/tobaccocontrol-2020-055874)
Supplement: Supplementary data [file tobaccocontrol-2020-055874supp001.pdf]

The impact of population tobacco control interventions on socioeconomic inequalities in smoking: a systematic review and appraisal of future research directions

Caroline Smith, Amanda Amos, Sarah Hill

Supplementary Data

**CONTENTS**	**PAGE**
S1. Search terms applied in MEDLINE	2-4
S2. Completed data extraction sheet	5-27
S3. Detailed equity impact findings	28-59
S4. Quality appraisal results	60-62

S1. Search terms applied in MEDLINE

Search No.	*Search Terms*
Search block: smoking	
1	exp smoking/
2	tobacco/ or tobacco, smokeless/ or nicotine/ or "tobacco use"/ or "tobacco use disorder"/ or smoking cessation/ or "tobacco use cessation"/ or tobacco smoke pollution/ or tobacco industry/
3	(smok$ or cigar$ or tobacco$).mp.
4	1 or 2 or 3
Search block: tobacco control interventions (non-cessation)	
5	(tobacco control adj3 (policy or policies or program$ or intervention$ or initiative$ or activity or activities or framework)).ti,ab.
6	((population level or population based or population orientated or population oriented) adj3 (policy or policies or program$ or intervention$ or prevention$ or project$)).ti,ab.
7	((smok$ or antismok$ or anti-smok$ or cigar$ or tobacco$) adj (policy or policies or program$)).ti,ab.
8	((cigar$ or tobacco$) adj3 (tax$ or excise or duty free or duty paid or customs or pric$)).ti,ab.
9	smoke-free policy/ or tobacco smoke pollution/lj, pc or air pollution/lj, pc or air pollution, Indoor/lj, pc
10	(tobacco control act or tobacco free or tobacco-free or smoke free or smoke-free or smokefree or (clean adj2 air)).ti,ab.
11	((reduc$ or prevent$) adj3 (environmental tobacco smoke or second hand smok$ or second-hand smok$ or secondhand smok$ or SHS or passive smok$ or involunt$ smok)).ti,ab.
12	((smok$ or antismok$ or anti-smok$ or cigar$ or tobacco$) adj3 (ban or bans or banned or prohibit$ or restrict$ or discourag$ or law or laws or bylaw$ or byelaw$ or by-law$ or bye-law$ or regulat$ or legislat$ or government$ or authorit$)).ti,ab.
13	(smok$ or antismok$ or anti-smok$ or cigar$ or tobacco$).ti,ab. and (product labeling/ or consumer product safety/ or advertising as topic/)
14	((smok$ or antismok$ or anti-smok$ or cigar$ or tobacco$) adj3 (market$ or campaign$ or advertis$ or advertiz$)).ti,ab.
15	((billboard$ or advertis$ or advertiz$ or sale or sales or sponsor$) adj3 (ban or bans or prohibit$ or restrict$ or limit$)).ti,ab.
16	((health or pictoral or pictorial or graphic$ or warn$) adj (warn$ or label$)).ti,ab.
17	((cigar$ or tobacco$) adj3 (pack or packet$ or packag$)).ti,ab.
18	(smok$ or antismok$ or anti-smok$ or cigar$ or tobacco$).ti,ab. and (exp mass media/ or internet/ or social media/ or social networking/)
19	((smok$ or antismok$ or anti-smok$ or cigar$ or tobacco$) adj3 (mass media or television or tv or radio or newspaper or film or movie or cinema or internet or online or net or web or www or mobile phone or cell phone or txt or pxt or sms or mms)).ti,ab.
20	(smok$ or antismok$ or anti-smok$ or cigar$ or tobacco$).ti,ab. and commerce/lj

Smith CE, *et al. Tob Control* 2021; 30:e87–e95. doi: 10.1136/tobaccocontrol-2020-055874

21	((retailer$ or vendor$ or tobacconist$ or merchant$) adj3 (educat$ or surveillance$ or prosecut$ or legislat$)).ti,ab.
22	(test purchas$ or voluntary agreement or point of sale or vending machine or contraband$ or smuggl$ or bootleg$ or cross border shopping).ti,ab.
23	(trade adj (restrict$ or agreement$)).ti,ab.
24	(retail$ or vend$ or tobacconist$ or merchan$ or purchas$ or sale or sales or store or stores$ or sell or selling or shop or shops).ti,ab. and (child/ or adolescent/ or minors/ or students/)
25	((retail$ or vend$ or tobacconist$ or merchan$ or purchas$ or sale or sales or store or stores or sell or selling or shop or shops) adj3 (child$ or adolescen$ or juvenile$ or girl$ or boy$ or teen$ or teens or minor$ or underage$ or under age$ or under-age$ or young people or young person or student$)).ti,ab.
26	(youth access adj3 restrict$).ti,ab.
27	((smok$ or antismok$ or anti-smok$ or cigar$ or tobacco$) adj3 (nationwide or nation wide or nation-wide or countrywide or country wide or country-wide or statewide or state wide or state-wide or citywide or city wide or city-wide or communit$)).ti,ab.
28	((smok$ or antismok$ or anti-smok$ or cigar$ or tobacco$) adj3 (public place$ or public space$ or public area$)).ti,ab.
29	((smok$ or antismok$ or anti-smok$ or cigar$ or tobacco$) adj3 (workplace or work place or work-place or worksite or work site or work-site or office$ or institution$)).ti,ab.
30	((smok$ or antismok$ or anti-smok$ or cigar$ or tobacco$) adj3 (hospital$ or clinic$ or prison$ or military$ or universit$ or college$ or school$)).ti,ab.
31	(communit$ adj3 (policy or policies or program$ or intervention$ or prevention$ or project$)).ti,ab.
32	5 or 6 or 7 or 8 or 9 or 10 or 11 or 12 or 13 or 14 or 15 or 16 or 17 or 18 or 19 or 20 or 21 or 22 or 23 or 24 or 25 or 26 or 27 or 28 or 29 or 30 or 31
Search block: tobacco control interventions (cessation)	
33	smoking cessation/ or "tobacco use cessation"/ or "tobacco use cessation products"/ or smoking/dt, pc, th
34	smoking cessation.kw.
35	(smoking and cessation).ti,ab.
36	cessation support.ti,ab.
37	((stop$ or quit$ or ceas$ or reduc$ or giv$ up) adj3 (smok$ or cigar$ or tobacco$ or nicotine$)).ti,ab.
38	((smok$ or cigar$ or tobacco$ or nicotine$) adj3 (cessation or abstinen$)).ti,ab.
39	((quit$ or cessation) adj (attempt$ or outcome$ or rate$ or relaps$ or maintain$)).ti,ab.
40	(quitline$ or quit line$ or quit-line$).ti,ab.
41	((smok$ or quit$ or tobacco$) adj3 (helpline$ or help line$ or help-line$ or ((telephone$ or phone$) adj counsel$))).ti,ab.
42	((smok$ or quit$ or tobacco$) adj3 (selfhelp or self help or self-help or manual$ or booklet$ or pamphlet$ or leaflet$ or letter$ or video$)).ti,ab.

Smith CE, *et al. Tob Control* 2021; 30:e87–e95. doi: 10.1136/tobaccocontrol-2020-055874

43	((smok$ or quit$ or tobacco$) adj3 (incentive$ or competition$ or contest$ or lotter$ or raffl$ or reward$ or prize$ or voucher$ or gift$ or inducement$ or contingent payment$ or deposit contract$)).ti,ab.
44	(quit adj2 win).ti,ab.
45	33 or 34 or 35 or 36 or 37 or 38 or 39 or 40 or 41 or 42 or 43 or 44
Search block: socioeconomic status	
46	exp socioeconomic factors/ or exp public assistance/ or exp social welfare/ or vulnerable populations/
47	(socioeconomic or socio economic or socio-economic or SES).ti,ab.
48	(social adj (class$ or group$ or grade$ or context$ or status)).ti,ab.
49	(social$ adj (disadvant$ or exclusion or excluded or depriv$)).ti,ab.
50	(townsend or carstairs or mosaic or health action zone$ or priority group$ or "index of multiple deprivation" or IMD or SIMD or WIMD or NIMDM).ti,ab.
51	(demographic$ or inequal$ or equity or depriv$ or disadvantag$ or disparit$ or poverty or poor or uninsur$ or insur$ or affluen$ or underserved or under served or under-served or high risk or high-risk or at risk or marginalised or marginalized).ti,ab.
52	(employ$ or unemploy$ or occupation$).ti,ab.
53	(income$ or educat$).ti,ab.
54	46 or 47 or 48 or 49 or 50 or 51 or 52 or 53
Search block: smoking + tobacco control interventions + socioeconomic status	
55	4 and (32 or 45)
56	54 and 55
57	limit 56 to (abstracts and english language and yr="2013-current")

S2. Completed data extraction sheet

	Intervention	Design	Location (time)	Sample	Smoking-related outcome	SES measures	Equity impact
Price/taxation increases (16)							
Bosdriesz (2016)	Tobacco price (domain-specific TCS score)	Repeat cross-sectional survey (EU-level) - 3 waves – combined with TCS for approximately same years as survey, recalibrated by authors to 2013 scoring system using published data	EU x 27 member states (2006-12)	73,617 adults (≥ 20)	Quit ratio (%)<br>Smoking intensity (no. per day)	Education	mixed/ unclear
Choi (2013)	Federal (national) tobacco excise tax increase introduced on 01/04/09	Cross-sectional survey (state-level) – using single wave of a wider longitudinal, cohort study involving adults who were current or former smokers in 2007	Minnesota, USA (2009)	727 adults (≥ 20) who reported being smokers on 01/04/09 – including those who had quit by the time of the 2009 survey (subsample of wider cohort study)	Tax increase helped think about quitting (y/n)<br>Tax increase helped cut down on cigarettes (y/n)<br>Tax increase helped make a quit attempt (y/n)	Education<br>Income	positive
Choi (2018)	State-level increase in tobacco excise and sales taxes in 2013	Cross-sectional survey (state-level), focusing on single wave after the tax increase	Minnesota, USA (2014)	1,229 past 30-day smokers, aged ≥ 18	Smoked fewer cigarettes to try and save money in past year (y/n)	Education<br>Household income	positive
Goldin (2013)	State-level cigarette excise tax (included in posted price) vs	Difference-in-difference analysis using data from an ongoing cross-	Multiple US states with a growing number of participating	1,288,031 adult survey respondents (≥ 18) of whom 274,137 were	Daily smoker (y/n)<br>Cigarette consumption (no. per day)	Income	mixed/ unclear

Smith CE, *et al. Tob Control* 2021; 30:e87–e95. doi: 10.1136/tobaccocontrol-2020-055874

	sales tax (levied at register)	sectional survey over a 17-year period (multiple states), combined with published data on cigarette excise and sales taxes	states such that national coverage achieved by 1994 (1984-2000)				
Havard (2018)	State-level increase (25%) in tobacco excise and customs duty (2010)	Interrupted time series analysis using birth records combined with hospital record data	New South Wales, Australia (2003-2011)	800,619 pregnancies among 534,513 women	Smoked at any point during pregnancy (y/n)	Socioeconomic index for areas (SEIFA) – index of relative socioeconomic disadvantage (IRSD)	mixed/ unclear
Hawkins (2014)	State-level cigarette tax increase (SF restaurant laws had no overall effect on maternal smoking)	Difference-in-difference analysis using maternal survey from national birth records, combined with published data on standardised, state-level cigarette taxes 9 months prior to birth	29 US states using original version of maternal survey from 2000-07 at least (2000-10)	17,699,534 births for which data on maternal smoking available	Maternal smoking during pregnancy (y/n) Smoking consumption (average number smoked daily)	Education	positive
Hu (2017)	Cigarette price index (cheapest and most popular price band)	Difference-in-difference analysis using cross-sectional health surveys (national-level) x 33 waves, combined with annual estimates of cigarette price (most popular and cheapest) and the non-price TC index for each country	Nine countries in Europe (1990-2007)	563,987 adults (aged 30-79)	Daily or occasional smoker (y/n)	Education Occupational group	positive

Smith CE, *et al. Tob Control* 2021; 30:e87–e95. doi: 10.1136/tobaccocontrol-2020-055874

Hummel (2015)	Cigarette price	Longitudinal survey (ITC national-level) – multiple waves per country (2 minimum)	France (2006, 2008 & 2012)<br>Germany (2007, 2009 & 2011)<br>Netherlands (2008-2013)<br>Ireland (2003, 2004 & 2006)<br>Scotland (2006 & 2007)<br>Rest of UK (2003-2008 & 2010)	Current smokers (aged ≥ 18), with between 461-1737 respondents per wave	Cigarette price led respondent to think about quitting in last 6 months (y/n)	Education<br>Income	positive
Keeler (2018)	Cigarette price	Cross-sectional survey (national-level) – two waves combined – merged with published data on standardised cigarette prices at the state-level	USA (2006-07 & 2010-11)	4,213 African Americans (≥ 18) who were recent active smokers i.e. current smoker or former smoker who quit less than 12 months ago	Quit intention (y/n)<br>Quit attempt in last 12 months (y/n)<br>Quit success – stopped for minimum of 3 months (y/n)	Household income	mixed/ unclear
MacLean (2016)	Cigarette tax	Longitudinal survey (national-level) with concurrent (cross-sectional) analysis of association between state-level cigarette tax (based on annual data from Tax Burden on Tobacco) and smoking behaviour	USA (1992-2008)	14,891 older adults aged ≥ 50 (119,935 person/years) – including 4,956 smokers (22,279 smoker/years)	Current smoker (y/n)<br>Cigarette consumption (average number smoked per day)	Education	mixed/ unclear
Mayne (2018)	Cigarette price	Longitudinal survey (study-specific) with time-to-event analysis of the	4 US cities (baseline: 1985-1986; follow-up: 1986-2001)	1,489 young adult smokers aged 18-30 at baseline (not pregnant)	Cessation attempt (y/n)<br>Sustained cessation to end of follow-up (y/n)	Education<br>Household income	mixed/ unclear

		association between smoking cessation and time-varying cigarette price (based on C2ER cost of living index for smallest geographical area available i.e. local, state or state)					
Parks (2017)	2013 cigarette tax increase (state-level)	Single wave of an ongoing cross-sectional survey (state-level) approximately 12 months after tax increase	Minnesota, USA (2014)	1,569 adults (≥ 18) who were either current smokers or recent quitters (up to 24 months before survey)	Self-reported smoking-related response to tax increase based on stages of change (no action, action, maintenance)	Composite index based on education and household income	positive
Regidor (2015)	Tobacco price	Repeat cross-sectional survey (national-level) – 8 waves – combined with consumer price index data on tobacco prices	Spain (1993-2012)	54,593 current and former smokers, aged 25-64	Age-standardised quit ratio (former smokers/ever smokers)	Education	mixed/ unclear
Vijayaraghavan (2013)	Cigarette price	Single wave of an ongoing cross-sectional survey (national-level)	USA (2006/07)	31,111 adults (≥ 18) who were either current smokers or recent quitters (up to 12 months prior to survey)	Cigarette consumption (average number smoked per day)<br>Quit for at least 90 days as time of survey (y/n)	Household income	mixed/ unclear
Yu (2018)	Cigarette price	Difference-in-difference analysis using data from ongoing cross-sectional survey	USA (1995-2009)	3,778,107 adults, aged ≥ 18	Current smoker (y/n)	Education	negative

Smith CE, *et al. Tob Control* 2021; 30:e87–e95. doi: 10.1136/tobaccocontrol-2020-055874

		(national-level) – 15 waves – combined with CDC data on cigarette taxes					
Zhang (2013)	Tobacco tax (state-level)	Ongoing cross-sectional survey (national and state-level), using data from four survey waves over a 12-year period, together with published data on state-level TC policies 2 years prior to survey	USA (1995-2007)	160,406 households containing children <18 years old	Complete home smoking ban (y/n)	Household education (highest educational level of parents in household)	positive
Smoke-free policies (17)							
Babb (2017)	Indoor, smoke-free workplace policies (including state-wide, local-level and voluntary bans)	Ongoing cross-sectional survey (national and state-level), using data from two waves 7-8 years apart	USA (baseline: 2003; follow-up: 2010-11)	145,477 employed adults (≥ 18) who work indoors at a single, primary non-residential site	Self-report SF workplace policy, covering both work areas and public/common indoor areas (y/n)	Occupational group	negative
Berg (2015)	Smoke-free building policies within multi-unit housing	Cross-sectional survey (study-specific) of participants in an online consumer panel with quota sampling to ensure adequate representation of racial/ethnic minorities, residents of south-eastern	USA (Jun – Jul 2013)	752 participants (18-65) in an online consumer panel who reside in multi-unit housing	Smoke-free building policy (complete/partial/none)	Education<br>Income	negative

		states, and recent tobacco users					
Bosdriesz (2016)	Smoke-free laws (domain-specific TCS score)	Repeat cross-sectional survey (EU-level) - 3 waves – combined with TCS for approximately same years as survey, recalibrated by authors to 2013 scoring system using published data	EU x 27 member states (2006-12)	73,617 adults (≥ 20)	Quit ratio (%)<br>Smoking intensity (no. per day)	Education	negative
Carton (2016)	Indoor smoking bans (state-level) covering workplaces, bars and/or restaurants	Scaled difference-in-difference analysis using data from repeat cross-sectional survey (national-level) – 15 waves – combined with data from multiple sources on state-level smoking legislation	USA (1996-2010)	4,049,706 adults, aged ≥ 18	Current smoker (y/n)	Education<br>Income	mixed/ unclear
Farley (2015)	Smoke-free unit policies in multi-unit housing, applying to all (100%) or some (partial) of the units in a building	Cross-sectional survey (study-specific) of random sample of MUH owners	New York City, USA – with city-wide smoke-free laws covering both indoor and outdoor public spaces (2012)	1,007 owners of multi-unit housing (3 or more units) in NYC	Smoke-free residential unit policy (100%/partial vs none)	Ownership of ≥1 certified low-income units	negative
Gentzke (2018)	Smoke-free building policies in multi-unit housing	Cross-sectional survey (study-specific), using baseline survey from	6 communities (3 x 2 pairs) across 3 US states – all of which subject	1,565 adult residents (≥ 18) of multi-unit housing	Live in smoke-free building (y/n)<br>SHS incursion in last 12 months (y/n)	Housing type (subsidized vs market-rate)<br>Education	mixed/ unclear

Smith CE, *et al. Tob Control* 2021; 30:e87–e95. doi: 10.1136/tobaccocontrol-2020-055874

		a wider longitudinal intervention study	to comprehensive SF laws covering public spaces at either a state or local-level (Aug – Dec 2012)				
Hood (2014)	Partial smoke-free park (county-level) policies	Cross-sectional, area-based analysis of 2 existing data sources: ANR tobacco control laws database and the American Community Survey (demographics)	USA (ANR: 2012; demographics: 2006-10)	3,143 US counties	County has designated SF park policy for some or all of its jurisdictions (y/n)	Area-based poverty (≥20% of county population living at or below federal poverty level)	negative
Huang (2015)	Local 100% smoke-free laws, covering restaurants, bars and/or workplaces (100% - smoking not allowed anywhere on premises and no size exemptions)	Cross-sectional, area-based analysis of 2 existing data sources: ANR tobacco control laws database and the American Community Survey (demographics)	US states which do not have a comprehensive SF law (state-level) but which encompass at least 8 areas covered by local laws (ANR: 2011; demographics: 2006-10)	14,298 localities in 10 eligible states: Alabama, Alaska, Indiana, Kentucky, Mississippi, Missouri, North Dakota, South Carolina, Texas, West Virginia	Local SF law coverage<br>• restaurants, bars and workplaces (y/n)<br>• restaurants, bars or workplaces (y/n)<br>• number of venue types covered (0-3)	Per capita income in locality<br>% local population (≥ 25) without high school diploma<br>% local population (≥ 16) who work in blue-collar jobs	mixed/ unclear
Hummel (2015)	Smoking restrictions in public places	Longitudinal survey (ITC national-level) – multiple waves per country (2 minimum)	France (2006, 2008 & 2012)<br>Germany (2007, 2009 & 2011)<br>Netherlands (2008-2013)<br>Ireland (2003, 2004 & 2006)<br>Scotland (2006 & 2007)	Current smokers (aged ≥ 18), with between 461-1737 respondents per wave	Restrictions led respondent to think about quitting in the last 6 months (y/n)	Education<br>Income	positive

Smith CE, *et al. Tob Control* 2021; 30:e87–e95. doi: 10.1136/tobaccocontrol-2020-055874

			Rest of UK (2003-2008 & 2010)				
Lidon-Moyano (2017)	Comprehensive, national smoke-free law, first introduced in 2005 (covering workplaces and indoor public spaces but with some exemptions for hospitality venues) and extended in 2010 to include all bars and restaurants as well as outdoor areas for some public buildings	Longitudinal survey, with the first wave conducted immediately prior to the 2005 ban and the second several years after the 2010 legislation	Barcelona, Spain (baseline: 2004-05; follow-up: May 13 – Feb 14)	1,245 adults (non-institutionalised), aged ≥ 16	Voluntary ban on smoking in home (complete, partial or none)	Education	negative
Murphy-Hoefer (2014)	Ban on smoking in vehicles carrying a person under the age of 16 (state-level)	Cross-sectional survey (national core module with additional state-level questions), using data from 4 consecutive waves from before and after the introduction of the SF vehicle law in 2008	Maine, USA (2007-10)	13,461 adults, aged ≥ 18	SF car rule (complete, when children present, none)<br>SF home rule (complete, partial, none)	Education<br>Income	mixed/ unclear
Platter (2018)	Smoke-free signage (unenforceable) in public, city parks	Pre/post observational study, involving two 6-week observational periods	Florida, USA (2011)	4 parks, containing 10 amenities (4 play-grounds, 4 picnic pavilions, 2 rest-rooms)<br>2,692 cigarette butts	Cigarette butts within boundary (25 ft) of city park amenities (number)	Neighbourhood income	mixed/ unclear

Regidor (2015)	National smoke-free legislation in 2006, covering workplaces, bars and restaurants	Repeat cross-sectional survey (national-level) – 8 waves	Spain (1993-2012)	54,593 current and former smokers, aged 25-64	Age-standardised quit ratio (former smokers/ever smokers)	Education	mixed/ unclear
Sandoval (2018)	Partial smoking ban in 2009 (state-level) covering a range of indoor public spaces – including hospitals, shopping malls and bars but excluding workplaces	Ongoing cross-sectional survey (state-level)	Geneva, Switzerland (1995-2014)	17,544 adults, aged 35-74	Smoking prevalence (%)<br>Quit ratio: ex-smokers/ever-smokers (%)	Education	negative
Schechter (2018)	Partial smoke-free legislation (state-wide) introduced in 2010, covering enclosed public spaces such as bars, restaurants and hotels	Cross-sectional questionnaire (study-specific), using baseline data from a wider prospective study	North Carolina, USA (2005-11)	668 pregnant non-smokers (≥ 18) attending pre-natal clinic who speak English &/or Spanish	SHS exposure based on cotinine levels at baseline assessment (n=<1ng/mL, y=1-3ng/mL)	Education	mixed/ unclear
Tchicaya (2016)	Comprehensive smoke-free legislation (national) introduced in 2006, covering workplaces, public transport and public spaces such as bars, restaurants and hotels	Cross-sectional survey (EU/national), using baseline data from three waves of a wider longitudinal study	Luxembourg (2005-08)	23,086 adults (≥ 16) who not resident in an institution	Smoking prevalence (% change, prevalence ratio, disparity index)<br>Quit due to SF law – former smokers in 2007 only (y/n)	Education<br>Household income	mixed/ unclear
Zhang (2013)	SF legislation score (state-level)	Ongoing cross-sectional survey (national and state-level), using data from four survey waves over a 12-	USA (1995-2007)	160,406 households containing children <18 years old	Complete home smoking ban (y/n)	Household education (highest educational level of parents in household)	positive

		year period, together with published data on state-level TC policies 2 years prior to survey					
Mass media campaigns (11)							
Bosdriesz (2016)	TC spending on public information campaigns (domain-specific TCS score)	Repeat cross-sectional survey (EU-level) - 3 waves – combined with TCS for approximately same years as survey, recalibrated by authors to 2013 scoring system using published data	EU x 27 member states (2006-12)	73,617 adults (≥ 20)	Quit ratio (%)<br>Smoking intensity (no. per day)	Education	negative
Durkin (2018)	Anti-tobacco TV adverts, covering different themes and emotional content: health effects (fear, sadness, combined negative); help-to-quit (hope)	Repeat cross-sectional survey (state-level) combined with advert exposure data (gross rating points) from two months prior to survey evaluation month for each participant	Victoria, Australia (Apr 12 – Nov 15)	7,658 smokers or past-month quitters who reported watching commercial TV on an average weekday	Quit attempt made in month prior to survey (y/n)	Socioeconomic index for areas (SEIFA) – index of relative socioeconomic disadvantage (IRSD)	mixed/ unclear
Havard (2018)	Pregnancy-specific anti-smoking adverts (May-June 2011)	Interrupted time series analysis using birth records combined with hospital record data	New South Wales, Australia (2003-2011)	800,619 pregnancies among 534,513 women	Smoked at any point during pregnancy (y/n)	Socioeconomic index for areas (SEIFA) – index of relative socioeconomic disadvantage (IRSD)	mixed/ unclear
Kim (2018)	Anti-smoking video-based public service	Between-subject, online randomised	USA (timescale not given)	136 current adult smokers, aged ≥ 18	Quit intentions (6-point scale)	Education	mixed/ unclear

Smith CE, *et al. Tob Control* 2021; 30:e87–e95. doi: 10.1136/tobaccocontrol-2020-055874

	announcements: stigmatising PSA vs non-stigmatising PSA (control)	experiment using video clips from real-world PSAs				Annual household income	
Lewis (2015)	Government-funded tobacco control TC campaigns, covering a range of themes including second-hand smoke	Repeat cross-sectional survey (national-level), combined with advert exposure data (gross rating points) from the same month as – and two months prior to – the survey date for each participant	England (Jan 04 – April 10)	9,872 households with at least one smoker aged ≥ 18	Smoke-free home (y /n)	Occupational group of head of household (NS-SEC)<br>Area-based deprivation (IMD)	mixed/ unclear
McAfee (2017)	Federally-funded, national tobacco education campaign (2013 Tips) via a range of media, with two ‘dose’ levels for the TV component: standard vs high (x3)	Randomised allocation of TV campaign dose across US television markets. Outcomes assessed via nationally representative, cross-sectional survey	USA (campaign: Mar – Jun 13; data collection: Jul – Oct 13)	5,733 adults (≥ 18) who were current smokers at the time of the 2013 Tips campaign launch – including those who subsequently quit successfully	Quit attempt lasting ≥ 1 day since launch of 2013 Tips campaign (y/n)	Education	mixed/ unclear
Neff (2016)	Federally-funded, national tobacco education campaign (2014 Tips, phase 2) delivered via a range of media	Longitudinal panel survey with pre/post analysis comparing pre-campaign (baseline) and immediate post-campaign (approx. 4 months after baseline) surveys	USA (campaign: Jul – Sep 14; data collection: Apr 14 – Jun 15)	4,248 adults (≥ 18) who were current smokers at the time of baseline survey and who completed both surveys	Quit attempt lasting ≥ 1 day in the last 3 months (y/n)<br>Intention to quit in next 30 days (y/n)<br>Intention to quit in next 6 months (y/n)	Education<br>[Income not included in pre/post analysis]	mixed/ unclear

Smith CE, *et al. Tob Control* 2021; 30:e87–e95. doi: 10.1136/tobaccocontrol-2020-055874

Nonnemaker (2014)	Anti-smoking TV adverts, grouped into those containing strong negative emotions/graphic images and other comparison	Repeat cross-sectional survey (state-level) combined with advert exposure data (gross rating points) from the past year (i.e. from the quarter in which the survey was conducted for each participant plus three previous quarters)	New York State, USA (2003-11)	9,408 adults (≥ 18) who were current smokers at the time of being surveyed – excludes those who quit successfully after exposure to TV adverts	Quit attempt lasting ≥ 1 day in the last 12 months (y/n)	Education<br>Income	mixed/ unclear
Rayens (2016)	Low-cost print media campaign on smoke-free, including loss-framed and gain-framed messages	Post-implementation, cross-sectional survey (involving residents from the intervention group of a wider community-based, smoke-free study)	Three rural communities in Kentucky, USA (campaign: started Nov 10 – Jan 11; data collection: Jul – Oct 11)	1,518 adults (≥ 18) who were resident in one of the three study counties	% of all adverts that led to a positive response for:<br>• recall<br>• like<br>• action – would prompt participant to contact local SF coalition<br>• made participant think<br>• affected participant emotionally	Education	mixed/ unclear
Vallone (2015)	National tobacco prevention truth campaign (ongoing) aimed at those aged 12-24. Delivered via range of media between 2000-07, with reduced online presence in 2012	Cross-sectional survey (single wave of ongoing longitudinal cohort study)	USA (Jul 12)	2,804 young adults, aged 24-34	Campaign awareness (y/n)	Education (individual and area-based)<br>Income (area-based)	mixed/ unclear
Zhang (2013)	Mass media TC campaigns (state-level)	Ongoing cross-sectional survey (national and state-	USA (1995-2007)	160,406 households containing children <18 years old	Complete home smoking ban (y/n)	Household education (highest educational	positive

		level), using data from four survey waves over a 12-year period, together with published data on state-level TC policies 2 years prior to survey				level of parents in household)	
Controls of advertising, promotion and marketing of tobacco (15)							
Bosdriesz (2016)	Advertising bans and health warning labels (domain-specific TCS scores)	Repeat cross-sectional survey (EU-level) - 3 waves – combined with TCS for approximately same years as survey, recalibrated by authors to 2013 scoring system using published data	EU x 27 member states (2006-12)	73,617 adults (≥ 20)	Quit ratio (%)<br>Smoking intensity (no. per day)	Education	negative
Czaplicki (2018)	Partial ban on sales of menthol cigarettes within 500ft of school	Cross-sectional audit	Chicago, USA (2017)	90 tobacco retailers located within 500ft of school	Tobacco retailer compliance with ban (y/n)	% residents in census tract living under poverty level	negative
Fry (2017)	Tobacco retailer notification scheme; in-store tobacco retail regulations (bans on advertising & PoS displays, restrictions on price labels/boars, display of warning notices)	Cross-sectional audit	New South Wales, Australia (Nov 2012 – Feb 2013)	1739 retailers across 8 (out of 10) NSW regions	Compliance with retailer notification scheme (y/n)<br>Compliance with in-store regulations (y/n)	Neighbourhood SES	negative
Gibson (2015)	Graphic warning labels vs text-only warning labels	Randomised online experiment	USA (2012)	2,285 current smokers, aged ≥ 26	Negative emotions (5-point scale averaged across 6 emotions)	Education	mixed/ unclear

					Perceived effectiveness (5-point scale averaged across 8 questions)<br>Intention to engage in avoidance behaviours (5-point scale)<br>Intention to hold back from smoking (5-point scale)<br>Intention to talk about warning (4-point scale)<br>Intention to talk to someone about quitting (4-point scale)<br>Intention to talk to medical professional about quitting (4-point scale)<br>Intention to quit (4-point scale)		
Giovenco (2018)	Ban on pharmacies selling tobacco	Empirical modelling study	New York City, USA (2017)	8,291 licensed tobacco retailers in 188 Neighbourhood Tabulation Areas	Tobacco retailer density (no. retailers per 1,000 residents)	Median income for NTA<br>% adults in NTA with less than high school education	negative
Hummel (2015)	Warning labels on cigarette packs	Longitudinal survey (ITC national-level) – multiple waves per country (2 minimum)	France (2006, 2008 & 2012)<br>Germany (2007, 2009 & 2011)<br>Netherlands (2008-2013)<br>Ireland (2003, 2004 & 2006)<br>Scotland (2006 & 2007)	Current smokers (aged ≥ 18), with between 461-1737 respondents per wave	Warning labels led respondent to think about quitting in the last 6 months (y/n)	Education<br>Income	positive

			Rest of UK (2003-2008 & 2010)				
Kuipers (2017)	Partial PoS display ban covering large shops introduced in April 2012	Interrupted time series analysis of ongoing cross-sectional survey (national-level), using data from 3 years before and after the ban was introduced	England (2009-2015)	129,957 adults (aged ≥ 18)	Current smoker (y/n)<br>Cigarette consumption (no. smoker per day)	Occupational group	mixed/ unclear
Lee (2015)	FDA tobacco advertising and labelling regulations (including bans on self-service displays, vending machines and sales of single cigarettes)	Cross-sectional audit	USA (Jan-Jul 2014)	33,543 advertising & labelling inspections of tobacco retailers – plus 718 warning letters	Advertising and labelling violation (y/n)	% adults in census tract with incomes under poverty line	mixed/ unclear
Moodie (2013)	Plain packaging vs own brand	Crossover RCT in naturalistic setting	Scotland x6 most populated cities (2011-12)	301 young adult (18-35) female smokers – of whom 187 completed the study and were included in analysis	Pack perceptions (5-point scale)<br>Pack feelings (5-point scale)<br>Feelings about smoking (5-point scale)<br>Warning salience & credibility (5-point scale)<br>Warning attention & processing (5-point scale)<br>Smoking/quit related behaviours x7 (mean no.)	Social grade	mixed/ unclear

Smith CE, *et al. Tob Control* 2021; 30:e87–e95. doi: 10.1136/tobaccocontrol-2020-055874

Nagelhout (2016)	Pictorial warning labels vs existing text warnings	Quasi-experimental study involving pre/post comparisons across 2 intervention and 2 control countries - using data from 2 waves of a longitudinal survey within each country (ITC Europe)	4 EU countries - 2 intervention: France (2008 & 2012) UK (2007/08 & 2010) 2 control: Germany (2007 & 2001) Netherlands (2008 & 2001)	6366 adult current smokers (aged ≥ 18) participated in baseline surveys – of whom 2863 took part in follow-up survey and reported still being smokers	Warning salience – noticed warning in past month (y/n) Cognitive response – made think about risks, made more likely to quit &/or led to think about quitting in past 6 months (y/n) Forgoing cigarettes as result of label (y/n) Avoidance of warnings (y/n)	Education	mixed/ unclear
Swayampakala (2018)	Pictorial warning labels plus pack inserts or plain packaging	Post-implementation evaluation using multiple waves of online longitudinal consumer panels – with replacement of quitters	Canada: inserts; Australia: plain packaging (2012-2014)	Current smokers (aged 18-64 years): Canada – 3153 smokers & 5826 observations; Australia – 2699 smokers & 5818 observations	Attention to HWLs (5-point scale) Cognitive responses to HWLs (9-point scale averaged across 3 questions) Forgoing cigarettes due to HWLs (y/n)	Education Income	mixed/ unclear
Thrasher (2015)	Pack inserts with pictorial warning labels vs previous text warnings	Post-implementation evaluation using multiple waves of online longitudinal consumer panels – with replacement of quitters	Canada (Sep 2012 – Jan 2014)	4,805 adult current smokers (aged 18-64 years)	Read pack inserts (y/n)	Education Income	mixed/ unclear
Thrasher (2018)	Pack inserts x 5 characteristic sets	Discrete choice experiment	USA (Nov 2016)	665 adult current smokers (18-50 years) – with around one-fifth excluded for not providing any preference data	Helpful for quitting (most/least) Motivating for quitting (most/east)	Education	mixed/ unclear

Tucker-Seeley (2016)	Voluntary ban by CVS pharmacies on selling tobacco; hypothetical ban on all pharmacies	Empirical modelling study	Rhode Island, USA (2015)	1,334 licensed tobacco retailers (including 135 pharmacies) in 240 census tracts	Tobacco retailer density (no. retailers per 10km of roadway in tract)	Median income for tract<br>% adults in tract with high school diploma or greater<br>% families in tract in poverty	neutral
Zacher (2014)	Plain packaging plus updated, larger pictorial warnings – introduced Oct 2012	Repeat cross-sectional observational study of patrons at outdoor venues	Melbourne & Adelaide – Australia (baseline: Oct 2011 – Apr 2012; follow-up: Oct 2012 – Apr 2013)	Convenience sample of 25 café strips in suburban areas (18 Melbourne, 7 Adelaide)	Packs observed per patron<br>Active smokers per patron<br>Packs observed per active smoker<br>Pack orientation (face-up, face-down, standing, concealed, external case, undetermined)	Socioeconomic index for areas (SEIFA) – index of relative socioeconomic disadvantage (IRSD)	mixed/ unclear
Population-level cessation support interventions (16)							
Benson (2016)	National system of reimbursement for smoking cessation pharmacotherapy (when used in combination with behavioural therapy) – implemented during 2011 only	Continuous cross-sectional survey (national-level) using data gathered over 4-year period	Netherlands (2009-12)	75,415 adults (≥ 15) – including 20,341 current smokers and recent quitters (since 2009)	Quit attempt using smoking cessation pharmacotherapy (y/n)	Education<br>Income	mixed/ unclear
Bosdriesz (2016)	Cessation support services (domain-specific TCS score)	Repeat cross-sectional survey (EU-level) - 3 waves – combined with TCS for approximately same years as survey, recalibrated by	EU x 27 member states (2006-12)	73,617 adults (≥ 20)	Quit ratio (%)<br>Smoking intensity (no. per day)	Education	mixed/ unclear

		authors to 2013 scoring system using published data					
Campbell (2014)	Provincial quitlines	Service monitoring data combined with national survey estimates of provincial smoking prevalence rates	Canada x 7 anonymised provinces (2007-09)	14,432 adult smokers (≥ 18) who had not received quitline counselling in past 12 months – from a total average annual population of 2,480,866 current smokers	Reach ratio (% quitline users in SES group / % current smokers in SES group)	Education	positive
Clare (2014)	Telephone quitline<br>Prescription for smoking cessation medication	Repeat cross-sectional survey (national-level), using data from 4 consecutive waves over a 10-year period	Australia (2001-10)	23,402 adult (≥ 18) current smokers and recent quitters	Use of quitline (y/n)<br>Use of prescribed cessation medication (y/n)<br>Quit in last 12 months (y/n)	Education<br>Income<br>Socioeconomic index for areas (SEIFA) – index of relative socioeconomic disadvantage (IRSD)	mixed/ unclear
Dhalwani (2014)	GP prescription of NRT during pregnancy (following a relaxation of prescribing rules to cover pregnant women)	Analysis of routinely collected GP-practice data (THIN database)	UK (2006-12)	Approximately 53,000 pregnant smokers of childbearing age (15-49) registered with THIN GP practice	NRT prescription (y/n)	Area-based (Townsend)	positive
Douglas (2013)	GP cessation advice and prescription for cessation medication (NRT, varenicline, bupropion)	Analysis of routinely collected GP-practice data (THIN database)	UK (Jul 2008 – Jun 2010)	460,938 adult (≥ 16) current smokers registered with THIN GP practice	Cessation advice recorded in GP notes (y/n)<br>Prescription for cessation medication – NRT, varenicline &/or bupropion (y/n)	Area-based (Townsend & Mosaic)	positive

Smith CE, *et al. Tob Control* 2021; 30:e87–e95. doi: 10.1136/tobaccocontrol-2020-055874

Hamilton (2016)	Local version of national financial incentives scheme – Quality & Outcomes Framework (QOF+) – for GPs introduced in July 2008	Pre/post evaluation using routinely collected data from electronic GP patient records	Hammersmith and Fulham, UK (Apr 2006 – Mar 2011)	13,785 current smokers (≥ 15) with no pre-existing conditions who registered with a participating GP practice	Cessation advice and/or SSS referral recorded in GP notes (y/n)	Area-based deprivation (IMD)	mixed/ unclear
Hardy (2014)	Cessation advice to pregnant smokers in general practice	Analysis of routinely-collected GP-practice data (THIN database)	UK (2006-09)	27,959 pregnant smokers of childbearing age (15-49) registered with THIN GP practice	Cessation advice recorded in GP notes (y/n)	Area-based (Townsend)	positive
Hummel (2015)	Free or low-cost stop smoking medication	Longitudinal survey (ITC national-level) – multiple waves per country (2 minimum)	France (2006, 2008 & 2012) Germany (2007, 2009 & 2011) Netherlands (2008-2013) Ireland (2003, 2004 & 2006) Scotland (2006 & 2007) Rest of UK (2003-2008 & 2010)	Current smokers (aged ≥ 18), with between 461-1737 respondents per wave	Free/low-cost medication led respondent to think about quitting in the last 6 months (y/n)	Education Income	positive
Molarius (2017)	Cessation advice during primary care consultation	Cross-sectional survey (study-specific) linked with demographic data from national registry	Sweden x 4 counties (2012)	30,188 adults (aged 16-84) – including 15,436 who had visited a primary care centre in last 3 months	Cessation advice during last primary care visit (y/n)	Education	positive
Nagelhout (2014)	National system of reimbursement for smoking cessation	Longitudinal survey with replenishment	Netherlands (2010-12)	Approximately 1900 respondents per wave who reported	Aware of reimbursement rules (4-point scale)	Household income	mixed/ unclear

Smith CE, *et al. Tob Control* 2021; 30:e87–e95. doi: 10.1136/tobaccocontrol-2020-055874

	medication (when used in combination with behavioural therapy) during 2011 – with awareness-raising campaign in Dec 10 to Jan 11	(ITC national-level) – 3 consecutive waves		smoking at previous wave	Aware of media campaign/ adverts for medications/ other media attention (4-point scale)<br>Quit attempt in last 6 months (y/n)<br>Used behavioural support in last 6 months (y/n)<br>Used cessation medication in last 6 months (y/n)<br>Quit success – still not smoking or back to smoking for < 1 month (y/n)		
Sadasivam (2013)	Web-assisted tobacco intervention (Decide2Quit), with referral via medical and dental practices (plus Google recruitment adverts)	Website registration database combined with 2010 data from an ongoing, cross-sectional survey (national-level)	USA (May 2010 – Jul 2011)	Website: 605 registered smokers<br>Survey: 69,992 adult smokers (≥ 18)	Website registration (y/n)<br>Website usage (no. web pages visited)	Education	negative
Schauer (2014)	Publicly-funded national network of free state quitlines	Cross-sectional survey (national-level) – single wave – combined with state-level estimates of TC spending per smoker	USA (Oct 2009 – Feb 10)	118,581 adults (aged ≥ 18)	Quitline awareness (y/n)<br>Quitline utilisation among current smokers and recent quitters (y/n)	Education<br>Income	mixed/ unclear
Skinner (2017)	Publicly-subsidised cessation medication (NRT, bupropion, varenicline)	Longitudinal cohort study (baseline, national-level survey) linked with claims data from	Australia (recruitment: 2006-09; follow-up: Dec 2011)	18,686 older adults (aged ≥ 45) who were regular smokers at baseline	Dispensing of subsidised prescription cessation medication by end of follow-up period (y/n)	Education<br>Socioeconomic index for areas (SEIFA) – index of relative	mixed/ unclear

		pharmaceutical and Medicare benefits schemes				socioeconomic disadvantage (IRSD)	
Taylor (2017)	GP prescription of varenicline or NRT	Prospective cohort study using routinely-collected GP-practice data (CPRD database)	UK (Sep 06 – Sep 15, including 4-year follow-up)	220,136 adult (≥ 18) smokers who were first time users of specified medication – and who had been registered with their GP practice for at least 12 months at time of prescription	Prescription (varenicline or NRT)<br>Quit smoking – assessed at 3/6/9 months and 1/2/4 years (y/n)	Area-based deprivation (IMD)	negative
Vaz (2017)	National network of NHS stop smoking services for pregnant women (SSSP)	Cross-sectional survey (study-specific) of SSSPs, combined with published data from 3 sources: routine SSSP performance monitoring data, hospital episode statistics and 2011 UK census	UK (2010-11)	121 of 141 SSSP – with multiple imputation of missing data for 18 further SSSP	Reach (% of pregnant smokers in SSSP who set quit date with SSSP help)<br>Effectiveness (% of pregnant smokers setting a quit date with SSSP support who remained abstinent after 4 weeks)	IMD for SSSP area<br>% people (≥ 16) with no qualifications within SSSP area<br>% people in managerial or professional occupations within SSSP area	mixed/ unclear
Other interventions (2)							
Hu (2016)	12-year English government strategy aimed at tackling health inequalities (initiated in 1999 and refined in 2003)	Modified difference-in-difference analysis using repeat cross-sectional surveys (national-level), drawing on 3 waves for each country approximately 10-years apart and	England with 3 control countries – Finland, the Netherlands and Italy (baseline 1989-92: pre-implementation: 1999-2000; post-implementation: 2009-10)	260,054 adults, aged 16-79 years	Current smoker (y/n)	Education (International Standard Classification)	negative

		covering broadly corresponding years					
Stafford (2014)	Area-based initiative aimed at tackling socioeconomic inequalities in health and in the social determinants of health implemented over 10-year period between 1998-2007 (New Deal for Communities)	Panel survey with replenishment (study-specific) – combined with repeat cross-sectional survey (national)	England (2002-2008)	28,982 adults from NDC intervention areas (19,574), NDC comparator areas (2014) or national survey (7,394)	Current smoker (y/n)	NDC intervention & comparator areas of high deprivation - plus Index of Multiple Deprivation for national survey	mixed/ unclear
Multiple policies (5)							
Bosdriesz (2015)	Range of national-level tobacco control policies introduced at various points but with initial increase in activity between 2000-02 – including smoke-free laws, tax increases and large public information campaigns	Ongoing cross-sectional survey (national-level) combined with TCS for corresponding years calculated by authors using a range of data sources	Netherlands (1988-2011)	259,140 ever smokers, aged ≥ 15	Quit ratio (%)<br>Smoking intensity (mean no. per day)	Education	mixed/ unclear
Bosdriesz (2016)	Combined TCS covering 6 policy domains	Repeat cross-sectional survey (EU-level) - 3 waves – combined with TCS for approximately same years as survey, recalibrated by authors to 2013	EU x 27 member states (2006-12)	73,617 adults (≥ 20)	Quit ratio (%)<br>Smoking intensity (no. per day)	Education<br>Occupational group	mixed/ unclear

Smith CE, *et al. Tob Control* 2021; 30:e87–e95. doi: 10.1136/tobaccocontrol-2020-055874

		scoring system using published data					
Havard (2018)	Graphic pack warnings plus extended partial smoking ban (2005/06)	Interrupted time series analysis using birth records combined with hospital record data	New South Wales, Australia (2003-2011)	800,619 pregnancies among 534,513 women	Smoked at any point during pregnancy (y/n)	Socioeconomic index for areas (SEIFA) – index of relative socioeconomic disadvantage (IRSD)	mixed/ unclear
Hu (2017)	Non-price TC index, covering 4 domains (smoke-free, bans on advertising and promotion, health warning labels, cessation services)	Difference-in-difference analysis using cross-sectional health surveys (national-level) x 33 waves, combined with annual estimates of cigarette price (most popular and cheapest) and the non-price TC index for each country	Nine countries in Europe (1990-2007)	563,987 adults (aged 30-79)	Daily or occasional smoker (y/n)	Education<br>Occupational group	mixed/ unclear
Pinilla (2017)	National tobacco control legislation in Spain (Law 28) – effective from 2006 and covering sales, supply, consumption and marketing of tobacco	Repeat cross-sectional survey (national-level), including retrospective data on smoking initiation and cessation in the previous 5 years - using 2 separate survey waves	Spain (2006-07 & 2011-12)	13,207 adult (≥ 21) smokers at start of 5-year period	Smoking cessation (y/n)	Education<br>Occupational group of head of household (NCO)	mixed/ unclear

S3. Detailed equity impact findings

Price/taxation increases (16 studies)

Bosdreisz (2016)	EU-wide analysis of the association between tobacco price (TCS domain score) and the quit ratio/ smoking intensity across 27 member states, combining 3 waves of an ongoing cross-sectional survey with published TCS data. Overall, no significant associations were found with price for either outcome (<u>quit ratio</u>: OR=0.99 (0.95, 1.04); <u>intensity</u>: β=0.17 (-0.10, 0.43)). Analyses of interactions between price and education indicated an equity negative effect in relation to the quit ratio (OR(high vs low)=1.06 (1.01, 1.10)). Differences in relation to smoking intensity were small and non-significant although relatively wide CIs suggest the need for some caution in concluding an equity neutral effect (β(high vs low)=-0.09 (-0.32, 0.14)).<br>*Comments:* Cross-sectional analysis makes it difficult to establish a causal relationship between TCS domain score and the quit ratio/smoking intensity. Minimal variation in tobacco prices over study period mean that the analyses primarily relate to between-country differences in price	mixed/unclear →<br>quit ratio = negative<br>intensity = neutral
Choi (2013)	Cross-sectional survey (state-level) of whether a US federal tobacco tax increase (2009) was perceived as being helpful in supporting cessation by smokers and recent quitters in Minnesota. Overall, 64.6% of smokers reported that the tax increase helped them to think about quitting, 46.7% that it helped them to reduce their cigarette consumption, and 29.0% that it helped them to make a quit attempt. There was clear evidence of a positive equity effect for education, with low education smokers (≤ high school) perceiving the increase to be more helpful than high education smokers (≥ college) across all outcome measures (think about quitting: OR=2.44 (1.57, 3.79); cut down: OR=2.18 (1.40, 3.42); quit attempt: OR=1.92 (1.17, 3.17)). In relation to income, there was also evidence of a positive equity effect among low (≤ $35k) compared to high income (≥ $75k) smokers for both cutting down (OR=1.47 (0.96, 2.24) i.e. did not quite reach statistical significance) and making a quit attempt (OR=1.64 (1.04, 2.59)). In contrast, the OR for thinking about quitting was much closer to 1 although wide CIs prevented us from drawing firm conclusions regarding equity impact.<br>*Comments:* Unclear whether outcome measure robust – retrospective, self-report that cigarette tax increase helped to think about quitting/reduce consumption/make quit attempt	positive
Choi (2018)	Post-implementation evaluation of a state-level increase in tobacco excise and sales taxes in Minnesota during 2013, using a single wave of an ongoing cross-sectional survey (state-level) to assess reductions in smoking consumption in the year following the tax increase. Overall, 69.8% of participants reported smoking fewer cigarettes in the past year to try and save money. There was evidence of an equity positive effect in relation to reduced consumption for both SES measures, with smokers from the lowest income groups being more likely to cut down than those from the highest income group (OR(<$25k vs ≥$75k)=2.83 (1.41, 5.70)). Findings were similar for education but did not quite reach statistical significance (OR(<high school vs ≥ college)=1.13 (0.90, 5.07)).<br>*Comments:* Focuses on current smokers only – those who quit successfully as a result of tax increase were excluded. Unclear whether outcome measure robust – self-report reduction in consumption to save money in	positive

	past year but not specifically linked to tax increase. Data not presented on reductions in smoking consumption prior to tax increase. Paper also looks at a range of other CEMS but these do relate to smoking prevalence or consumption	
Goldin (2013)	Difference-in-difference analysis of the impact of state-level cigarette taxes (excise and sales) on smoking prevalence and cigarette consumption in the USA, using data from an ongoing cross-sectional survey over a 17-year period combined with published data on cigarette taxes. Overall, a 1% increase in excise taxes (i.e. those included in the posted price) led to 0.117% (p<0.01) reduction in smoking prevalence and 0.341% (p<0.01) reduction in cigarette consumption. Equivalent estimates for sales taxes (i.e. those levied at the register) were non-significant although of a similar order for smoking prevalence (a 1% tax increase led to 0.132% decrease in prevalence). Sales taxes appeared to have minimal impact on consumption but the standard error was large (β=-0.022%, SE=0.290). Tests of interaction indicated a differential tax response by SES for sales but not excise taxes. Among low income participants, an increase in sales taxes led to much larger reduction in both prevalence and consumption than among higher income groups (<u>prevalence</u>: β=-0.501, p<0.01; <u>consumption:</u> β=-1.379, p<0.01), indicating an equity positive effect. In contrast, parameter estimates for excise taxes were close to zero (albeit again with relatively large SEs), suggesting an equity neutral effect.	mixed/unclear → excise tax = neutral sales tax = positive
Havard (2018)	Pre/post evaluation of the impact of a state-level increase in tobacco excise and customs duty (25%) during 2010 on smoking during pregnancy in New South Wales (Australia), using an interrupted time series analysis (segmented regression) of birth records combined with hospital admissions data. Smoking during pregnancy fell from 17.1% in June 2003 to 10.6% in December 2011, representing a 0.39% relative monthly decrease in prevalence over the study period. Stratified analyses (with CIs) were presented by SES but differences by area deprivation were not formally tested. Across the whole study period, the rate of decline decreased with increasing deprivation (<u>low</u>=-0.43% (-0.48%, -0.37%); <u>mid</u>=-0.36% (-0.32%, -0.40%); <u>high</u>=-0.20% (-0.24%, -0.16%)). Overall, there was a non-significant further reduction in the rate of decline following the tax increase (change in trend = -0.17% (-0.62%, +0.29%)). While this enhancement of the downward trend was seen in both the low and high-deprivation groups (<u>low:</u> change in trend = -0.93% (-1.78%, -0.07%); <u>high:</u> change = -0.90% (-1.55%, -0.25%)), there was a non-significant dampening of the trend in the mid-deprivation group (change = 0.33% (-0.28%, 0.94%). *Comments:* Paper also looks at a number of other interventions not included in this review as they had no effect either overall or by SES (i.e. exposure to general anti-smoking TV adverts and a complete SF ban)	mixed/unclear → non-linear SES effect
Hawkins (2014)	Difference-in-difference analysis of the impact of state-level cigarette tax increases during pregnancy on maternal smoking and cigarette consumption across 29 US states, combining national birth records with published, monthly data on cigarette taxes (standardised). Overall, there was a significant negative association between cigarette tax at the time of conception and maternal smoking, such that a $1 increase in taxes was associated with a 0.50% decrease in smoking prevalence and a 0.32 decrease in daily cigarette consumption. There was also a significant interaction between maternal education and cigarette tax for both outcomes.	positive

	Among the lowest education group (≤ 11 years), there was a 1.02% decrease in smoking prevalence (p=0.001) and a 0.62 decrease in daily consumption (p=0.07). In contrast, among the highest education group (≥ 16years), there a 0.03% decrease in prevalence (p=0.7) and a 0.01 decrease in consumption (p=0.7).<br>*Comments:* Restricted to 29 states using the original maternal survey from national birth records so may not be generalisable to other population groups. Reliance on self-report smoking data from national birth record may reduce reliability of outcomes. Analyses were also presented by racial/ethnic group – showing that the decrease in maternal smoking among the lowest education group was limited to white and black women, with only minimal changes being seen among other racial/ethnic groups. Study additionally looked at the impact of SF restaurant laws but no overall association was found so analyses were not presented by SES	
Hu (2017)	Difference-in-difference analysis of the relationship between smoking prevalence and country-level cigarette price indices (cheapest and most popular price category) across 9 European countries during 1990-2007, using 33 waves of national-level, cross-sectional health surveys. Overall, there was a significant decline in smoking prevalence with increasing cigarette price among both men (most popular: OR=0.86 (0.74, 0.99)) and women (cheapest: OR=0.50 (0.34, 0.75)). While a non-significant decline was also seen among men for the cheapest cigarette price (OR=0.89 (0.63, 1.26)), there did not appear to be an association between smoking and the most popular price category among women (OR=1.04 (0.67, 1.63)) but CIs were relatively wide. Analyses by education and occupation found that ORs were typically lower in the low SES groups across both price measures (with the exception of the popular price index in women) although tests of interaction were non-significant – perhaps reflecting the wide CIs. For example, the largest difference for men was in the popular price index by education (low: OR=0.85 (0.70, 1.03)); high: OR=1.15 (0.81, 1.64)) and for women was in the cheapest price index for education (low: OR=0.55 (0.37, 0.82); high: OR=0.89 (0.55, 1.45)).<br>*Comments:* Focuses on concurrent cigarette price and does not explore lag effects	possibly positive
Hummel (2015)	Analysis of policy triggers (cigarette price) for thinking about quitting among current smokers in six European countries (France, Germany, the Netherlands, Ireland, Scotland and the rest of the UK), using multiple waves of the ITC Europe (national-level) longitudinal surveys. Overall, between 59-74% of respondents mentioned that cigarette price had triggered thoughts of quitting. There was broadly consistent evidence of an equity positive (or possibly positive) effect, with low income smokers being more likely to report cigarette price as a trigger than high income smokers across all six countries, and low education smokers being more likely to report price as a trigger in three countries. ORs (low vs high) for education were closer to one in the other three countries (Netherlands: OR=0.95 (0.80, 1.13); Ireland: OR=0.96 (0.70, 1.30); UK: OR=1.03 (0.89, 1.18)) but relatively wide CIs meant that it was not possible to draw firm conclusions about equity impact.<br>*Comments:* Focuses on current smokers only – those who quit successfully as a result of cigarette price were excluded. Unclear whether outcome measure robust – retrospective, self-report that policy triggered thoughts of quitting in last 6 months	positive
Keeler (2018)	Cross-sectional survey (national-level) of the association between cigarette prices and quit intentions, attempts and success among African American, recent active smokers (including those who quit within the last 12	mixed/unclear →

	months) in the USA, combining two survey waves with published data on standardised cigarette prices at the state-level. Overall, there was a significant positive association between current cigarette prices and quit intentions (OR=2.55 (1.56, 4.15)). There was also some evidence of a positive association between cigarette prices (lagged by 12 months) and quit attempts (OR=1.44 (0.87, 2.37)), as well as of negative association between lagged prices and quit success (OR=0.78 (0.25, 2.37)), but neither of these associations were statistically significant. Analyses by household income suggested a positive equity effect in relation to quit intentions/attempts and a negative equity effect in relation to quit success although the findings were not definitive. In stratified analyses, the only significant effect was for quit intentions among those from low-income households (OR=3.51 (1.75, 7.02)). The OR for quit intentions among high-income respondents was lower but CIs were wide and overlapped those for the low-income group (OR=1.94 (0.95, 3.96)). There was a similar pattern of results in relation to quit attempts, although SES differences were smaller (OR(low)=1.79 (0.90, 3.54); OR(high)=1.19 (0.57, 2.46)), and a reverse pattern in relation to quit success (OR(low)=0.54 (0.08, 3.43); OR(high)=1.00 (0.26, 3.90)). Interactions between price effects and income level were non-significant – perhaps reflecting the wide CIs.<br>*Comments:* Cross-sectional analysis of association between price and quit behaviours with a reliance of retrospective, self-report data	quit intentions/ attempts = possibly positive<br>quit success = possibly negative
MacLean (2016)	Analysis of the association between state-level cigarette taxes and smoking behaviour (prevalence and daily consumption) in the USA, using data from a longitudinal survey (national-level) of adults aged 50 or over combined with annual tax data from Tax Burden on Tobacco, with a focus on concurrent (cross-sectional) associations adjusted for state-level and secular trends. Overall, a $1 tax increase was associated with a 2.93% (SE=1.87%; p>0.10) non-significant increase in current smoking but a 0.6457 (SE=0.3105; p<0.05) decrease in daily cigarette consumption (based on model 2 estimates which appear to match model specification used in SES analysis). Stratified analyses (with CIs) were presented by SES but differences by education were not formally tested. The absolute increase in smoking prevalence per $1 increase in cigarette tax was nearly identical across the two education groups (< high school = 3.58% (SE=3.42%; p>0.1); ≥ high school = 3.41% (SE=2.04%; p<0.1)) although this does suggest a smaller relative increase in the low education group given the higher underlying prevalence. A larger decrease in consumption was seen among the low education group but this did not appear to be significant (< high school = 1.3041 (SE=0.8567; p>0.1); ≥ high school = 0.4880 (SE=0.3030; p<0.1)).<br>*Comments:* Concurrent (cross-sectional) analysis of association between tax and smoking behaviour – does not explore lag effects. Direction of effect for prevalence varied across model specifications with parameter estimates ranging from -0.8% decrease to +2.93% increase, suggesting that these findings might be less robust	mixed/unclear →<br>prevalence = mixed/unclear (dependent on model specification)<br>consumption = possibly positive
Mayne (2018)	Longitudinal survey (study-specific) of the association between cigarette prices and quit attempts among young adult (18-30) smokers from US four cities, using a time-to-event analysis, and combining data on local cigarette prices (time-varying for smallest geography available) from the C2ER cost of living index. Overall, a $1 increase in the price of a cigarette carton (10 packs) was associated with a 16% in the likelihood of making a quit attempt	mixed/unclear →<br>quit attempts = positive

Smith CE, *et al. Tob Control* 2021; 30:e87–e95. doi: 10.1136/tobaccocontrol-2020-055874

	and an 8% in the likelihood of making a sustained quit attempt. In relation to quit attempts, there was a significant interaction between household income and price, with a higher likelihood of making an attempt in the lowest income group (low: HR=1.23 (1.12, 1.34); medium: HR=1.14 (1.05, 1.24); high: HR=1.12 (1.04, 1.20); p(interaction)=0.04). The reverse pattern was seen in relation to sustained quitting although here the interaction was non-significant (low: HR=1.04 (0.88, 1.22); medium: HR=1.02 (0.92, 1.13); high: HR=1.15 (1.06, 1.25); p(interaction)=0.3). For education, the likelihood of making both a quit attempt and a sustained quit attempt was higher in the lowest education group although neither interaction was statistically significant (quit attempt – <high school: HR=1.32 (1.10, 1.58); high school: HR=1.13 (1.04, 1.22); some college: HR=1.15 (1.07, 1.23); ≥degree: HR=1.15 (1.05, 1.25); p(interaction)=0.3; sustained quit attempt – <high school: HR=1.39 (1.00, 1.93); high school: HR=1.05 (0.92, 1.19); some college: HR=1.04 (0.95, 1.13); ≥degree: HR=1.11 (1.01, 1.22); p(interaction)=0.8).<br>*Comments:* Restricted to four US cities so may not be generalisable to other population groups	sustained quit attempts = mixed/unclear (varies by SES)
Parks (2017)	Cross-sectional survey (state-level) exploring socioeconomic differences in self-reported responses to a $1.75 cigarette tax increase in Minnesota during 2013 among current smokers and recent quitters, using a single wave of an ongoing population survey. Overall, 41.8% of respondents reported no action as a result of the tax increase, 37.8% an action-oriented change (reduced consumption or quit attempt), and 20.5% quit maintenance. Medium/high SES respondents were less likely to report taking action than low SES respondents (low SES – no action=35%, action=43%, maintenance=22%; medium/high SES – no action=48%, action=33%, maintenance=19%; OR(medium/high vs low)=0.66 (0.50, 0.87)). [A latent class analysis found similar results with minimal differences in % of 'tax-driven quitters' across SES groups but almost double the rate of 'responsive daily smokers' (i.e. quit attempt, reduce consumption and/or price minimisation behaviours) in low SES respondents.]<br>*Comments:* Unclear whether outcome measure robust – retrospective, self-report responses to tax increase approximately 12 months after introduction. Representative at the state-level but unlikely to be generalisable to other population groups	positive
Regidor (2015)	Analysis of the association between tobacco prices and the quit ratio among adults (25-64 years) over a 20-year period in Spain, using 8 waves of a cross-sectional survey (national-level) combined with consumer price index data on tobacco prices. Stratified analyses (with p-values but not CIs) were presented by SES and gender but differences were not formally tested. After adjusting for secular trends and the introduction of smoke-free legislation, there was no clear overall relationship between tobacco prices and the quit ratio. In males, parameter estimates were non-significant for both SES groups but there was some evidence of a decreasing quit ratio with increasing tobacco prices in the low education group (low SES: β=-0.060, p=0.116; high SES: β=-0.001, p=0.982), suggesting an equity negative effect. In females, there was a significant decline in the quit ratio among low educated women (β=-0.089, p=0.002) but a greater, albeit non-significant, decline among high educated women (β=-0.106, p=0.123).	mixed/unclear → males = possibly negative<br>females = mixed/ unclear (lack of power in high SES)

Vijayaraghavan (2013)	Cross-sectional survey (national-level) of the association between state-level, average pack-price (terciles based on self-report data of the price paid) and smoking behaviours (daily consumption and 90-day quit success) among current smokers and recent quitters in the USA, using a single wave of an ongoing population survey. Stratified analyses (with p-values but not precise estimates of CIs) of smoking behaviour by education or pack-price were presented but the interaction between these two variables was not formally tested. Overall, cigarette consumption was lower in higher pack-price states (highest tercile=12.8% vs lowest tercile=16.3%, p<0.001). Within each of the four income levels groups, cigarette consumption decreased with increasing pack-price (p(trend)<0.001 in all cases). In high and intermediate pack-price states, consumption appeared to increase slightly with increasing income (high: p(trend)=0.054; intermediate: p(trend)<0.001), but little variation was seen by income level in low pack-price states (p(trend)=0.3). Among the lowest and highest income groups, differences in quit success rates by pack-price appeared to be minimal although the lack of precise estimates and relatively wide CIs made it difficult to draw firm conclusions. Differences were larger in the middle-income groups, with lower quit success rates in the lowest pack-price states. Within each of the three pack-price groups, quit success rates increased with increasing income (p(trend)<0.001 in all cases).<br>*Comments:* Cross-sectional analysis of association between cigarette price and smoking/quitting behaviours with a reliance on self-report data in relation to both exposure (price) and outcomes. Unclear how continuing abstainers who quit less than 90 days before the survey are handled (censored or counted as unsuccessful quitter?)	mixed/unclear → SES interaction not formally tested; precise parameter estimates not given (consumption); non-linear SES effect (quit success)
Yu (2018)	Difference-in-difference analysis of the impact of state-level cigarette tax on smoking prevalence in the USA, using data from 15 waves of an ongoing cross-sectional survey (national-level) combined with CDC data on cigarette taxes. The overall effect size was small but statistically significant, with a $0.68 increase in tax being associated with a 0.25% (SE=0.09%) decrease in smoking prevalence. Stratified analyses (with standard errors) were presented by educational level but SES differences were not formally tested. There was a significant decrease in smoking prevalence with increasing cigarette taxes among the high education group (β=-0.27%, SE=0.09%, p=0.001) but a smaller non-significant decrease among the low education group (β=-0.09%, SE=0.15%, p=0.568) – but CIs appear to be overlapping suggesting that these SES differences were non-significant	possibly negative
Zhang (2013)	Analysis of the association between state-level tobacco taxes and complete home smoking bans in households containing underage children (<18 years) in the USA, combining data from multiple waves of a cross-sectional survey (national and state-level) with published data on tobacco taxes 2 years prior to each survey. Among non-smoking households, there was a significant interaction between education and tobacco tax, such that educational disparities in home smoking bans decreased with increasing tax (OR(high vs low education; per $ increase in tax)=0.48 (0.38, 0.61)). This interaction continued to be significant in models adjusting for other policy measures i.e. SF legislation and mass media TC campaigns (OR(high vs low education; per $ increase in tax)=0.53 (0.42, 0.66)). Among smoking households, the interaction was similarly significant in the single policy analysis (OR(high vs low education; per $ increase in tax)=0.72 (0.55, 0.96)) but not in the multi-policy analysis.	positive

	Here though, the increase in home smoking bans still appeared to be greater in lower educated households (low education: OR(per $ increase in tax)=1.14 (0.87, 1.49); high education: OR(per $ increase in tax)=0.98 (0.96, 1.26)).<br>*Comments:* Cross-sectional analysis (even with lag period) makes it difficult to establish a causal relationship between state-level tobacco taxes and the prevalence of home smoking bans	
Smoke-free policies (17 studies)		
Babb (2017)	Trend analysis of SF workplace coverage among indoor workers in the USA, using two waves of an ongoing cross-sectional survey (national and state-level). Overall, the proportion of workers who reported being covered by a SF policy increased significantly between 2003 (77.7 (77.2, 78.1)) and 2010-11 (82.8 (82.4, 83.2)). There were marked SES differences in SF coverage across occupational groups at the time of the baseline survey (white-collar: 82.0 (81.5, 82.4); blue-collar: 62.6 (61.1, 64.0); service: 68.1 (67.1, 69.2)). While these differences persisted to some extent at follow-up, there was nevertheless a distinct narrowing of the occupational gap (white-collar: 84.9 (84.5, 85.3); blue-collar: 74.3 (72.9, 75.7); service: 78.9 (77.8, 78.9)).<br>*Comments:* Looks at self-report SF coverage in the workplace but amalgamates a variety of different policies, including state-wide, local-level and voluntary workplace bans. Analysis does compare changes in SF coverage across states with/without comprehensive state-level SF laws but not by SES	negative
Berg (2015)	Cross-sectional survey (study-specific) of smoke-free building coverage among multi-unit housing residents in the USA, using a quota sample of participants in an online consumer panel. Overall, 16.2% of residents reported having a complete SF policy in their housing unit, 27.5% reported having a partial policy, and 56.3% having no policy. Within this, there was evidence of a negative equity effect, with the likelihood of **not** having a complete policy decreasing with increasing levels of education (β=−0.68 (−1.11 to −0.26)) and increasing levels of income (β=−0.77 (−1.36 to −0.18)).<br>*Comments:* Data collection via online consumer panel with over-representation of racial/ethnic minorities, residents of south-eastern states, and recent tobacco users which may limit generalisability	negative
Bosdreisz (2016)	EU-wide analysis of the association between smoke-free laws (TCS domain score) and the quit ratio/ smoking intensity across 27 member states, combining 3 waves of an ongoing cross-sectional survey with published TCS data. Overall, there was significant negative association between SF laws and smoking intensity (β=-0.15 (-0.27, -0.03)) but no such association was found for the quit ratio (OR=0.99 (0.97, 1.02)). Analyses of interactions between SF laws and education indicated an equity negative effect in relation to both the quit ratio (OR(high vs low)=1.06 (1.03, 1.08)) and smoking intensity (β(high vs low)=-0.17 (-0.31, -0.03)).<br>*Comments:* Cross-sectional analysis makes it difficult to establish a causal relationship between TCS domain score and the quit ratio/smoking intensity	negative
Carton (2016)	Scaled difference-in-difference analysis (additionally adjusted for the year in which the ban was introduced) of the impact of state-level, indoor smoking bans (covering workplaces, restaurants and/or bars) on adult smoking	mixed/unclear →

	prevalence in the USA, using 15 waves of an ongoing cross-sectional survey (national-level) combined with data from multiple sources on smoke-free legislation. Overall, comprehensive SF laws covering all three venue types had a small but significant effect on smoking rates, being associated with a 0.7% (SE=0.2%) decrease in prevalence – compared to an average annual (absolute) decrease of approximately 0.4% over the study period. A complex pattern of results was reported by SES, involving a large number of significance tests (36), where small effect sizes made it difficult to interpret the findings in relation to equity impact. Stratified analyses (with standard errors) were presented by income (8 levels) and education (4 levels) but differences were not formally tested. The largest SES differences were found for income in relation to bans covering bars and restaurants which led to a 5.7% (SE=1.8%) decrease in smoking prevalence among those earning <$10k but more minimal changes among those earning >$15k (range: -0.9% to +0.3%), possibly suggesting an equity positive effect. No other SES differences appeared to be significant (based on the standard errors) but parameter estimates were not sufficiently precise to rule an equity effect. There was, for example, some suggestion that restaurant-only and workplace bans may be less effective in the lowest income groups. Differences in relation to education were typically smaller but followed a similar pattern.<br>*Comments:* Multiple comparisons (36) increase the likelihood of spurious statistically significant findings	lack of power; complex pattern
Farley (2015)	Cross-sectional survey (study-specific) of multi-unit housing owners in New York City (USA) on the adoption of smoke-free policies within their residential units. Overall, 33% of owners had such a policy in place, with the majority of these (83%) reporting a 100% ban on smoking in all units within the building. Bivariate analyses found that owners with no certified low-income units (high SES group) were more likely to have a smoke-free unit policy than those with one or more such units (36% vs 26%, p<0.01). While this difference was no longer significant in multivariate analyses (OR=0.93 (0.65, 1.34)), CIs were wide and unadjusted analyses may be more appropriate as they provide estimates of actual policy coverage across SES groups (Giovenco, 2018).<br>*Comments:* Low SES group (ownership of ≥1 certified low-income units) is likely to include market-rate units but data on the proportion of such units is not available	negative
Gentzke (2018)	Cross-sectional analysis of smoke-free building coverage and its association with SHS incursions among multi-unit housing residents across 6 states in the USA, using the baseline survey from a wider longitudinal study of a community-based intervention. Overall, just under a quarter (23.4%) of residents reported living in a SF building, with a further 9.3% living in building where smoking was prohibited in shared areas. Multivariate analyses suggested that the likelihood of living in a SF building was slightly higher for subsidized than for market-rate residents (OR=1.06 (0.78, 1.43)) although the wide CIs meant it was not possible draw firm conclusions. There was, however, evidence of significant positive equity effect in relation to education, with the highest educational group (graduate school) being less likely to be covered by a SF building policy than those with less than a high school education (OR=0.53 (0.29, 0.97)). While SHS incursions were less common among those living in a SF building (OR=0.59 (0.44, 0.80)), this difference was only apparent for market-rate (OR=0.50 (0.35, 0.71)) and not for subsidized residents (OR=0.91 (0.53, 1.58)), suggesting an equity negative effect in relation to adherence although overlapping CIs indicate that the difference was not significant.	mixed/unclear → coverage = positive; incursions = possibly negative

	Comments: Study restricted to 6 communities in 3 states and may not be generalisable to other population groups	
Hood (2014)	Cross-sectional, area-based analysis of smoke-free park policy (complete or partial) coverage in the USA, combining data from two existing sources (ANR tobacco control laws database and the American Community Survey). Overall, 11.3% (355) of US counties had a SF park policy covering at least one jurisdiction. Multivariate analyses demonstrated that low-SES counties (i.e. those where ≥20% of population live at or below federal poverty level) were less likely to have a SF park policy than high-SES counties (OR=0.32 (0.23, 0.45)), indicating an equity negative effect.<br>*Comments:* A validation of the ANR database, covering 10% of counties, found that 9% (25) of counties that were listed as not having a SF park policy did in fact have such a policy. When restricted to the revised validation sample, multivariate analyses showed that the direction of the SES effect was reversed and, while this finding was non-significant (p=0.92), it does suggest a need for caution in interpreting the results of the full sample	negative
Huang (2015)	Cross-sectional, area-based analysis of local-level 100% SF law coverage (restaurant, bar and/or workplace) across 10 states in the USA where there was no existing state-level ban, combining data from two existing sources (ANR tobacco control laws database and the American Community Survey). Overall, local SF coverage was low in 9 out of 10 states, with the percentage of localities covered ranging from 0.5% to 2%, and the average number of SF law types ranging from 0.01 to 0.48. The one exception was West Virginia where 37% of localities were covered by all three SF law types and 81% were covered by at least one law type (with an average number of laws per locality of 1.74). Analyses were presented separately for each state, SES measure and outcome, giving a total of 90 comparisons. In relation to education, there was broadly consistent evidence of an equity negative effect (13 comparisons indicated a statistically significant negative effect, 13 were non-significant but suggestive of a negative effect, and 4 were inconclusive with ORs close to 1 but wide CIs). For occupation, the evidence again pointed to an equity negative effect in 7 out of 10 states (10 comparisons were significantly negative, 8 were non-significant but in the direction of a negative effect, and 3 were inconclusive). In the remaining three states, results were non-significant but suggestive of an equity positive effect for all three outcome measures. In relation to income, 25 comparisons indicated an equity negative effect (10 significant). Here, though an equity positive effect was evident in West Virginia across all outcome measures, with a statistically significant effect for the number of law types (OR=0.572 (0.336, 0.973)). The authors suggest that patterns of local SF coverage may evolve over time, with an initial widening of inequalities in states where the process of adopting such laws is in its infancy, followed by a narrowing of SES differences as local SF laws become more established and widespread.<br>*Comments:* Multiple comparisons (90) increase the likelihood of spurious statistically significant findings	mixed/unclear → largely negative but possibly positive in some areas for income & occupation
Hummel (2015)	Analysis of policy triggers (smoking restrictions in public places) for thinking about quitting among current smokers in six European countries (France, Germany, the Netherlands, Ireland, Scotland and the rest of the UK), using multiple waves of the ITC Europe (national-level) longitudinal surveys. Overall, between 29-55% of	positive

	respondents mentioned that smoking restrictions had triggered thoughts of quitting. There was broadly consistent evidence of an equity positive (or possibly positive) effect, with low income and low education smokers being more likely to report smoking restrictions as a trigger than high SES smokers in three countries (France, Germany and Scotland). ORs (low vs high) were closer to one in the other three countries (and possibly negative for education in Ireland) but relatively wide CIs meant that it was not possible to draw firm conclusions about equity impact.<br>*Comments:* No information on the strength of the smoking restrictions in each country (i.e. whether ban comprehensive or partial). Focuses on current smokers only – those who quit successfully as a result of smoking restrictions were excluded. Unclear whether outcome measure robust – retrospective, self-report that policy triggered thoughts of quitting in last 6 months	
Lidon-Moyano (2017)	Pre/post evaluation of comprehensive, national smoke-free legislation (2005/2010) in Spain, using a longitudinal survey to assess changes in the voluntary adoption of smoke-free homes (SFH), with a specific focus on Barcelona. Overall, there was a significant increase in the proportion of SFH, rising from 55.6% in the pre-legislation survey (2004-05) to 72.6% in the post-legislation survey (2013-14), giving an adjusted pre/post prevalence ratio (PR) of 1.30 (1.20, 1.41). This increase was primarily driven by the adoption of complete SFH (PR=1.61 (1.35, 1.93)) as opposed to partial SFH (PR=1.07 (0.91, 1.26)). Prior to the ban, differences in SFH adoption by educational group were minimal (low=56.4%, intermediate=56.9%, high=53.9%). While improvements were seen across all SES groups, the largest increases were found in the high education group (PR=1.41 (1.23, 1.60)), with smaller increases being reported for the low (PR=1.25 (1.10, 1.43)) and intermediate groups (PR=1.21 (1.05, 1.41)). Although suggestive of an equity negative effect, overlapping CIs prevent us from drawing any firm conclusions.<br>*Comments:* Amalgamation of the initial and follow-up legislation into a single intervention with an extended implementation period of 6 years – and the absence of a control – makes it difficult to attribute the observed changes to the SF legislation rather than to secular trends	possibly negative
Murphy-Hoefer (2014)	Pre/post evaluation of 2008 legislation banning smoking in vehicles carrying a child in the US state of Maine, using four waves of the BRFSS survey. Overall, the proportion of participants reporting a complete ban on smoking in their car increased significantly (p=0.004) from 74.9% in 2007 (pre-ban) to 78.8% in 2008-10 (post-ban). This increase was evident across all education and income levels, with the largest increases seen among the lowest SES groups. For example, among those with less than a high school diploma the increase was 13.4% (p=0.30) compared to 3.8% (p=0.05) among those with ≥4 years of college education, and among those with an annual income of <$20k the increase was 14.2% (p=0.05) compared to 2.8% (p=0.27) among those with an income of ≥$75k. A formal statistical analysis was not undertaken, however, of the interaction between the intervention and the SES measures, making it difficult to draw any firm conclusions about the equity effect. Similarly, the overall proportion of participants reporting a complete ban on smoking in their home increased significantly (p=0.009) from 79.9% pre-ban to 83.1% post-ban. SES differences were, though, smaller and the direction of effect less clear cut. For education, the “less than high school group” again showed the largest	mixed/unclear →<br>SF cars = possibly positive<br>SF homes = mixed/ unclear (SES effect varies/non-linear)

	increase of 7.7% (p=0.49) but the "some college education" group demonstrated a similar level of increase at 6.2% (p=0.05). In contrast, the largest increases by income were seen for those earning between $50-75k (9.1%, p=0.009) and those earning $20-35k (5.6%, p=0.21). Again, no formal test of interaction was presented.<br>*Comments:* In the absence of a control, the increase in SF cars cannot be directly attributed to the new legislation – the findings may be explained by secular trends and/or concomitant policy initiatives	
Platter (2018)	Pre/post observational study of smoke-free signage (unenforceable) across four public city parks in Florida (USA) during 2011, using discarded cigarette butts within park amenities (10) as a proxy for smoking. Overall, there was a significant decrease (p=0.028) in the number of cigarette butts found within the 10 park amenities following signage placement. While no formal analysis of SES differences was presented, the two parks with the highest median neighbourhood income demonstrated the largest percentage decreases in the number of butts found. In the two lower income parks, there was a slight overall fall in cigarette butts but an increase in three out of the five amenities.<br>*Comments:* The small sample size, coupled with a lack of formal statistical analyses by SES, makes it difficult to draw any firm conclusions. Adequacy of cigarette butts as a proxy measure for smoking is unclear. Restricted to a small sample of city parks in Florida so unlikely to be generalisable to other locations	mixed/unclear → small sample; descriptive analysis only
Regidor (2015)	Pre/post evaluation of the impact of national smoking-free legislation in Spain (introduced in 2006) on the quit ratio among adult ever-smokers (25-64 years), using 8 waves of a cross-sectional survey (national-level) from 1993-2012. Stratified analyses (with p-values but not CIs) were presented by SES and gender but differences were not formally tested. Prior to the ban, there was a significant upward trend in the quit ratio across each of the four strata (males – low SES: trend=0.71 (p=0.001); high SES: trend=0.67 (p=0.007); females – low SES: trend=0.51 (p=0.002); high SES: trend=1.17 (p=0.003)). Following the ban, there was an immediate step-change increase in all groups, with the largest increases being seen among females and low education smokers, suggesting a possible equity positive effect (males – low SES: step-change=1.93 (p=0.264); high SES: step-change=1.40 (p=0.540); females – low SES: step-change=4.66 (p=0.001); high SES: step-change=3.10 (p=0.365)). Over the next five years, however, differential changes in the previous upward trend led to an apparent widening of inequalities (males – low SES: trend-change=-0.79 (p=0.023) i.e. upward trend levelled out ; high SES: trend-change=0.02 (p=0.956) i.e. upward trend continued; females – low SES: trend-change=-0.83 (p=0.009) i.e. upward trend reversed; high SES: trend-change=-1.19 (p=0.082) i.e. upward trend levelled out). Taken together, the step-change and trend-change were estimated to have a negative impact on the 2012 quit ratio (compared to the quit ratio that would have been observed if the legislation had not been introduced) in three of the four strata, with the overall equity impact being negative for males (low SES=-1.8; high SES=0.0) but potentially positive for females (low SES=-2.0; high SES=-2.7).	mixed/unclear →<br>step-change = possibly positive<br>trend = possibly negative<br>overall = mixed/ unclear (varies by sex)
Sandoval (2018)	Pre/post evaluation of a partial smoking ban in Geneva (Switzerland) from 2009 which covered a range of public indoor spaces but excluded workplaces, using data from an ongoing cross-sectional survey between 1995-2014. Overall, the ban was associated with a step-change decrease in smoking prevalence (β=-0.043 (-0.073, -0.014)) and a corresponding step-change increase in the quit ratio (β=0.059 (0.009, 0.108)). There was no significant	negative

	change in the underlying trends, although the two measures did gradually return to their pre-ban levels, suggesting a possible reversal of the previous improving trends. Smoking prevalence demonstrated a marked widening of SES inequalities after the ban, using both the relative index of inequality to compare the lowest educational group with the highest (RII(pre)=1.84 (1.60, 2.11), RII(post)=3.01 (2.27, 3.99), p(RII change)<0.01), and the equivalent absolute measure – the slope index of inequality (SII(pre)=0.14 (0.11, 0.16), SII(post)=0.19 (0.15, 0.24), p(SII change)=0.03). The quit ratio similarly showed a widening of SES inequalities although here differences were only significant for the absolute measure: RII(pre)=0.76 (0.68, 0.86), RII(post)=0.65 (0.53, 0.81), p(RII change)=0.05 (widely overlapping CIs suggest p-value may be incorrect); SII(pre)=-0.15 (-0.20, -0.11), SII(post)=-0.27 (-0.35, -0.19), p(SII change)=0.02.	
Schechter (2018)	Pre/post evaluation of the impact of a state-wide, partial smoking ban in North Carolina (USA), introduced in 2010 and covering public indoor spaces such as bars, restaurants and hotels, on SHS exposure among pregnant non-smokers, using baseline data from a wider prospective study. Overall, there was a significant decrease in cotinine levels following the introduction of the ban (before: mean=0.78, SD=0.60; after: mean=0.52, SD=0.55; p<0.001), although a regression discontinuity analysis suggested that the decline was steepest in the period immediately prior to the ban being implemented and then levelled out to some degree. Pre/post differences across educational groups were not formally analysed although descriptive statistics were presented. These data suggest that absolute inequalities may have narrowed slightly while relative inequalities have remained broadly similar but it is not possible to be certain based on the analyses presented (before: no college=0.95 (SD=0.64), any college=0.71 (SD=0.58); after: no college=0.63 (SD=0.67), any college=0.47 (SD=0.49)).	mixed/unclear → descriptive analysis only
Tchicaya (2016)	Pre/post evaluation of the impact of national smoke-free legislation (2006) on smoking prevalence and smoking cessation in Luxembourg, using three waves of a cross-sectional (EU/national) survey between 2005-08. Overall, there was a significant decline in smoking prevalence following the ban, falling from 23.1% in 2005 to 17.9% in 2008 (% change=-22.5 (-27.3, -17.4), p<0.001). This decline in prevalence was seen across all educational groups and all income levels but there was no clear direction of effect and CIs were wide. For example, in relation to education, the percentage change ranged from -23.2 (-31.6, -13.7) in those with a primary education only, to -19.3 (-25.8, -12.3) in those with a secondary education, and -27.8 (-39.1%, -14.3) in those with a tertiary education. While prevalence ratios (a measure of relative inequality across the lowest vs highest SES levels) and the disparity index (a combined, summary measure of inequality across all SES levels) were used to explore equity effects in more detail, the pattern of results was inconsistent and inconclusive. Here, there was some evidence of a negative equity effect in relation to education among males, with the prevalence ratio (PR) increasing from 2.1 (1.8, 2.5) in 2005 to 2.6 (2.1, 3.2) in 2008 and the disparity index (DI) increasing from 19.6% to 24.6%. For household income, the DI again showed an increase from 17.0% to 23.0% but PRs remained stable (2005: 2.0 (1.7, 2,3); 2008: 2.0 (1.7, 2.4)). Pre/post differences were minimal across all four measures for women. In all analyses, however, CIs were relatively wide (or not available in the case of DI), making it difficult to draw any firm conclusions about the intervention equity effect in terms of smoking	mixed/unclear → smoking prevalence = mixed/unclear (complex pattern)<br>quit as result of ban = positive

	prevalence. Clearer SES differences did emerge in relation to the proportion of former smokers in 2007 who reported quitting due (at least in part) to the 2006 smoking ban: OR(primary vs tertiary education)=1.85 (1.08, 3.15); OR(lowest vs highest income quartile)=2.14 (1.21, 3.80).<br>*Comments:* The validity of the smoking cessation outcome measure is unclear – different groups may respond differently to the question of whether they stopped smoking as a result of the ban. Changes in prevalence may reflect secular trends or the effects of other concomitant policy changes but, in the absence of a control group or a formal time series analysis, these possible alternative explanations cannot be assessed	
Zhang (2013)	Analysis of the association between state-level SF laws score and complete home smoking bans in households containing underage children (<18 years) in the USA, combining data from multiple waves of a cross-sectional survey (national and state-level) with published data on SF laws 2 years prior to each survey. Among non-smoking households, there was a significant interaction between education and the SF score, such that educational disparities in home smoking bans decreased with increasing SF score (OR(high vs low education; high vs low SF)=0.67 (0.67, 0.93)). This interaction was no longer significant in models adjusting for other policy measures i.e. cigarette tax and mass media TC campaigns (<u>low education</u>: OR(high vs low SF)=1.19 (0.79, 1.78); <u>high education</u>: OR(high vs low SF)=1.14 (0.67, 1.93)). Among smoking households, the interaction between education and SF score was non-significant although the increase in home smoking bans with increasing SF score did appear to be greater in lower educated households (<u>low education</u>: OR(high vs low SF)=1.97 (1.09, 3.55); <u>high education</u>: OR(high vs low SF)=1.38 (0.72, 2.64)). These differences again disappeared in models adjusted for other policy measures.<br>*Comments:* Cross-sectional analysis (even with lag period) makes it difficult to establish a causal relationship between state-level SF laws and the prevalence of home smoking bans	positive
Mass media campaigns (11 studies)		
Bosdreisz (2016)	EU-wide analysis of the association between public health campaigns (TCS domain score) and the quit ratio/smoking intensity across 27 member states, combining 3 waves of an ongoing cross-sectional survey with published TCS data. Overall, no significant associations were found with the campaign score for either outcome (<u>quit ratio</u>: OR=1.00 (0.96, 1.04); <u>intensity</u>: β=-0.02 (-0.24, 0.20)). Analyses of interactions between the campaign score and education indicated an equity negative effect in relation to the quit ratio (OR(high vs low)=1.06 (1.03, 1.09)) and possibly also smoking intensity although here the difference did not quite reach statistical significance (β(high vs low)=-0.14 (-0.32, 0.04)).<br>*Comments:* Cross-sectional analysis makes it difficult to establish a causal relationship between TCS domain score and the quit ratio/smoking intensity	negative
Durkin (2018)	Analysis (combining data from a cross-sectional survey with TV advert exposure data) of the relationship between the degree of exposure to anti-tobacco TV adverts and past-month quit attempts in Victoria (Australia). Overall, exposure to adverts containing either fear-based or combined negative emotional content had a positive	mixed/unclear → complex pattern

	linear effect on quit attempts. Results for sadness-evoking and hope-evoking adverts were non-significant although CIs were wide and it was not possible to rule out an effect, particularly in relation to hope. The interaction between exposure to combined negative content and SES was significant (p=0.045), with the effect being apparent in low SES areas only (equity positive). All other two-way interactions with SES were non-significant but wide CIs again make it difficult to draw firm conclusions (equity unclear but possibly positive for fear and possibly negative for sadness). The three-way interaction between fear, hope and SES was significant (p=0.003), such that the effect of fear-evoking adverts increased with greater levels of exposure to hope-evoking content among high (p=0.041) but not low (p=0.955) SES groups (equity negative). A significant three-way interaction was also seen for sadness, hope and SES (p=0.031). Here, the effects of sadness appeared to increase with greater exposure to hope-based content in low SES areas (p=0.376) but to decrease in high SES areas (p=0.282) although neither of these stratified analyses were statistically significant (unclear but possibly positive). The three-way interaction between combined negative content, hope and SES was non-significant but ORs and CIs were not supplied (equity unclear).	
Havard (2018)	Pre/post evaluation of the impact of pregnancy-specific, anti-smoking adverts on smoking during pregnancy in New South Wales (Australia), using an interrupted time series analysis (segmented regression) of birth records combined with hospital admissions data. Smoking during pregnancy fell from 17.1% in June 2003 to 10.6% in December 2011, representing a -0.39% relative monthly decrease in prevalence over the study period. Stratified analyses (with CIs) were presented by SES but differences by area deprivation were not formally tested. Across the whole study period, the rate of decline decreased with increasing deprivation (low=-0.43% (-0.48%, -0.37%); mid=-0.36% (-0.32%, -0.40%); high=-0.20% (-0.24%, -0.16%)). Overall, there was a non-significant dampening of the downward trend following the anti-tobacco campaign in May-June 2011 (change in trend = +0.27% (-0.84%, +1.38%)). While this attenuation of the trend was seen in both the low and particularly the high-deprivation groups (low: change in trend = +0.66% (-1.47%, +2.83%); high: change = +1.77% (+0.13%, +3.43%)), there was a non-significant enhancement of the downward trend in the mid-deprivation group (change = -0.55% (-2.0%, +0.92%).<br>*Comments:* Paper also looks at a number of other interventions not included in this review as they had no effect either overall or by SES i.e. exposure to general anti-smoking TV adverts and a complete SF ban)	mixed/unclear → non-linear SES effect
Kim (2018)	Randomised experiment among current smokers in the USA comparing the effects of anti-smoking public service announcements (PSAs) containing stigmatising vs non-stigmatising content on quit intentions. Overall, exposure to the stigmatising PSA had a larger positive effect on quit intentions than the non-stigmatising, control PSA. There was a significant interaction between PSA type and income level (β=0.62, p<0.01), such that low-income participants were less likely to intend quitting than high-income participants if they viewed the stigmatising PSA (equity negative) but more likely to intend quitting if they viewed the non-stigmatising PSA (equity positive). This effect appeared to be partly driven by reduced feelings of shame among low income/low self-efficacy participants following exposure to the stigmatising PSA. The interaction between PSA type and	mixed/unclear → varies by SES

	education level was non-significant (β=0.09, p=0.63) and, while CIs were not supplied, the closeness of the β estimate to zero may suggest an equity neutral effect although it is not possible to be sure.	
Lewis (2015)	Analysis (combining data from a repeat cross-sectional survey with TV advert exposure data) of the relationship between the degree of exposure to tobacco control adverts (total and SHS-specific) and current smoke-free home status in England. Overall, there was no association between smoke-free home status and total advert exposure (concurrent month: OR=1.01 (0.99, 1.04); 1-month lag: OR=1.00 (0.98, 1.02); 2-month lag: OR=1.00 (0.98, 1.02)). SHS-specific campaigns were relatively uncommon, being aired in only 12 out of 75 months. Nevertheless, there was a significant positive association between smoke-free home status and exposure to SHS adverts one month earlier (OR=1.07 (1.01, 1.13)) although no such associations were found for SHS advert exposure in the same month (OR=0.99 (0.93, 1.05)) or two months earlier (OR=0.98 (0.92, 1.04)). Interactions between SES (both measures) and advert exposure (total and SHS-specific) were non-significant at all lags, leading the authors to conclude that there was no evidence of widening socioeconomic inequalities (equity neutral), although they did highlight the low statistical power associated with these interaction analyses. As parameter estimates and CIs were not supplied, it was not possible to make an independent assessment of power.<br>*Comments:* Only looks at the direct effect of TV adverts on smoke-free home status and does not assess the indirect effect resulting from a reduction in smoking prevalence	mixed/unclear → lack of power
McAfee (2017)	Randomised evaluation of 2013 Tips tobacco education campaign in the USA which was delivered via a range of platforms (TV, radio, billboards, print media and a website), with the level of TV coverage being manipulated across (geographically-based) television markets such that two-thirds received a standard dose of 800 nationally-aired GRPs and one-third received an additional 1600 locally-aired GRPs. While the study as whole examined a number of different outcomes, the SES analysis focused on quit attempts since the launch of the 2013 Tips campaign. Overall, the proportion of current smokers making a quit attempt following the campaign launch was higher among the high-dose than the standard-dose markets (OR=1.20, p=0.029). The interaction between market dose and education was not formally tested but rather a stratified analysis was undertaken, with ORs for high-dose vs standard-dose markets being presented separately for each education level (CIs not supplied). Descriptive statistics for the standard-dose intervention suggest an equity negative effect: quit attempt rates were higher among college graduates (40.6%) than among those with some college education (33.9%) and those with a high school education or less (32.9). Equivalent statistics for the high-dose intervention suggest a differential effect across education groups, with an increase in quit attempt rates among those with some college education (45.4%) but minimal change among those a high school education or less (32.4%) and college graduates (42.6%). Thus, the high-dose intervention still appears to be equity negative, with the lowest education group having lower quit attempt rates than the other two groups. These findings are reflected in the stratified ORs for high-dose vs standard-dose market where only the OR for some college education was statistically significant (OR=1.60, p<0.001). In comparison with the standard-dose intervention, therefore, the	mixed/unclear → non-linear SES effect

	high-dose intervention appears to have a mixed equity effect, narrowing inequalities between the middle and high education groups, but widening them between the middle and low groups.<br>*Comments:* The 20 largest US television markets were excluded from the study due to cost considerations.	
Neff (2016)	Pre/post evaluation of the 2014 Tips (phase 2) tobacco education campaign in the USA, using two consecutive waves of a longitudinal panel survey of current smokers at baseline. Overall, the proportion of smokers making a quit attempt increased following the campaign (OR=1.17, p=0.03), as did the proportion intending to quit within the next 30 days (OR=1.26, p=0.08) and within the next 6 months (OR=1.28, p=0.01). There was some evidence of a differential intervention effect in relation to education but the lack of formal statistical testing of interactions, coupled with wide CIs in the stratified analyses, made it difficult to draw firm conclusions. Following the campaign, quit attempts appeared to fall among those with less than a high school education (OR=0.87 (0.58, 1.28)), to remain broadly stable among high school graduates (OR=1.06 (0.83, 1.36)), and to increase among those with some college education (OR=1.23 (1.00, 1.52)) or with a college degree (OR=1.22 (0.87, 1.70)). Similar results were found in relation to quit intentions for all but the lowest education group (less than high school) where large pre/post increases were seen, albeit with very wide CIs: OR(quit within 30 days)=2.12 (0.73, 6.11), OR(quit within 6 months)=1.62 (0.80, 3.29).<br>*Comments:* Definition of quit attempts inconsistent over the pre/post-implementation periods: baseline analysis appears to include current smokers only whereas the post-implementation analysis also includes recent quitters. Possible latency effects from phase 1 of 2014 campaign (aired from Apr-Jul).	mixed/unclear → attempts = possibly negative<br>intentions = mixed/ unclear (non-linear SES effect)
Nonnemaker (2014)	Analysis (combining data from a cross-sectional survey with TV advert exposure data) of the relationship between exposure to anti-smoking TV adverts and past-year quit attempts in New York State. Advert exposure was assessed using two measures – GRPs and self-reported recall – and divided according to those that contained emotional/graphic content and others. There was a significant positive association between exposure to anti-smoking adverts and past-year quit attempts across all measures of exposure (with the exception of GRPs for comparison adverts where the results were inconclusive) and across all SES groups (low/high education, low/high income). While interactions between exposure and SES were not formally tested, a stratified analysis (including CIs) was presented. In relation to education, there was some evidence that self-recalled emotional/graphic adverts may be more effective among low SES (high school graduate or less) than high SES (some college or more) smokers but the difference was non-significant: OR(low)=1.61 (1.21, 2.15), OR(high)=1.34 (1.01, 1.77). Results for other exposure measures were closer although relatively wide CIs prevented us from drawing firm conclusions about equity imapct. In the relation to income, ORs were higher for low SES (<$30,000) than high SES smokers across all exposure measures but again relatively wide, overlapping CIs made it difficult to distinguish between an equity positive and an equity neutral effect. The largest differences were seen for emotional/graphic GRPs (OR(low)=1.62 (1.18, 2.21), OR(high)=1.38 (1.12, 1.70)) and for emotional/graphic self-recalled exposure (OR(low)=1.73 (1.24, 2.42), OR(high)=1.47 (1.11, 1.96)).<br>*Comments:* Focuses on current smokers only – participants who quit successfully were excluded even though exposed to adverts	mixed/unclear → emotional/graphic content = possibly positive<br>other content (GRP) = mixed/unclear (varies by SES)<br>all content (self-report) = neutral

Rayens (2016)	Post-implementation evaluation of a low-cost, print media campaign around smoke-free, using a single cross-sectional survey of residents from three rural Kentucky communities in the USA to compare advert recall and perceived effectiveness (like, action, think, emotion) for loss-framed and gain-framed messages. There was a significant negative association between advert recall and education (high school diploma or not) for gain-framed messages: the low education group recalled 11.1% fewer adverts than the higher education group (p<0.001). SES differences for loss-framed messages were minimal, with recall being 0.4% higher in the low education group (p=ns). Perceived effectiveness outcomes were typically better in the low education than the higher education group although these differences only reached statistical significance for loss-framed messages: action-loss=+16.06% for low vs high SES, p=0.002; action-gain=+9.60%, p=ns; think-loss=+8.53%, p=0.023; think-gain=-0.79%, p=ns; emotion-loss=+12.57%, p=0.01; emotion-gain=+11.33%, p=ns. Differences in relation to the like outcome were smaller and non-significant for both loss-framed (+3.58 for the low education group) and gain-framed (+1.66%) messages. CIs were not supplied but the relatively high parameter estimates associated with the non-significant findings are indicative of a lack of power.	mixed/unclear → recall (gain) = negative recall (loss) = neutral effectiveness/like = positive
Vallone (2015)	Evaluation of the ongoing, national tobacco prevention campaign aimed at young people (12-24 years) in the USA, using a single wave (cross-sectional) of a longitudinal cohort study. During 2000-07, the campaign was delivered across a range of media platforms (including television, radio, billboards and online) but in 2012, the year of study, the campaign was limited to online outlets (website, social media and online adverts). The study explored levels of campaign awareness among those aged 24-34, a group with at least some exposure to the full campaign in 2000-07, as well as ongoing exposure to the online campaign in 2012. The pattern of results varied somewhat by age. Among the 24-29 age group, there was a significant negative association between campaign awareness and individual-level education, such that those in the lowest education group (less than high school) had much lower rates of campaign awareness than college graduates: OR=0.18 (0.06, 0.54). Analyses using area-based measures of SES, in contrast, suggested a non-linear effect – CIs were though wide and non-significant (e.g. for education: OR (quartile 2 vs 1)=1.54 (0.89, 2.67); OR(quartile 3 vs 1)=0.90 (0.49, 1.65; OR(quartile 4 vs 1)=1.23 (0.63, 2.39)). The reverse pattern was, however, apparent among the 30-34 age group, with a non-linear effect being seen in relation to individual-level education but a possibly negative trend being seen for the two area-based SES measures.<br>*Comments:* Not possible to untangle effects of current and past campaign exposure	mixed/unclear → varies by SES
Zhang (2013)	Analysis of the association between state-level mass media TC campaigns and complete home smoking bans in households containing underage children (<18 years) in the USA, combining data from multiple waves of a cross-sectional survey (national and state-level) with published data on mess media campaigns 2 years prior to each survey. Among non-smoking households, there was a significant interaction between education and mass media campaigns, such that educational disparities in home smoking bans decreased in the presence of such campaigns (OR(high vs low education; campaigns y/n =0.80 (0.71, 0.91)). While this interaction was no longer significant in models adjusting for other policy measures (i.e. cigarette tax and mass media TC campaigns), it was not possible to rule out an equity positive effect (<u>low education</u>: OR(campaigns y/n)=1.42 (1.19, 1.71); <u>high</u>	positive

	education: OR(campaigns y/n)=1.32 (1.03, 1.70)). Among smoking households, the interaction was similarly significant in both the single policy analysis (OR(high vs low education; campaigns y/n)=0.71 (0.61, 0.83)) and the multi-policy analysis (OR(high vs low education; campaigns y/n)=0.76 (0.65, 0.89)).<br>*Comments:* Cross-sectional analysis (even with lag period) makes it difficult to establish a causal relationship between state-level mass media TC campaigns and the prevalence of home smoking bans	
Controls on advertising, promotion and marketing of tobacco (15 studies)		
Bosdreisz (2016)	EU-wide analysis of the association between advertising bans/health warning labels (TCS domain score) and the quit ratio/ smoking intensity across 27 member states, combining 3 waves of an ongoing cross-sectional survey with published TCS data. In relation to advertising bans, no overall significant associations were found for either outcome (quit ratio: OR=1.03 (0.99, 1.07); intensity: β=0.11 (-0.09, 0.30)). Analyses of interactions between such bans and education indicated an equity negative effect in relation to the quit ratio (OR(high vs low)=1.06 (1.02, 1.10)) and possibly also smoking intensity although here the difference did not quite reach statistical significance (β(high vs low)=-0.19 (-0.38, 0.01)). In relation to health warning labels, no overall significant associations were again found for either outcome (quit ratio: OR=0.99 (0.93, 1.06); intensity: β=-0.31 (-0.64, 0.03)). Here, analyses of interactions between HWLs and education were suggestive of an equity negative effect in relation to the quit ratio (OR(high vs low)=1.07 (0.98, 1.17)) and possibly also smoking intensity (β(high vs low)=-0.35 (-0.83, 0.13)) although neither of these differences reached statistical significance<br>*Comments:* Cross-sectional analysis makes it difficult to establish a causal relationship between TCS domain score and the quit ratio/smoking intensity	possibly negative
Czaplicki (2018)	Cross-sectional audit of tobacco retailer compliance with a partial ban on sales of menthol cigarettes within 500 feet of a high school in Chicago. Overall compliance was relatively low (57%) possibly reflecting the partial nature of the ban. In unadjusted analysis, there was some evidence of lower compliance in areas with higher levels of poverty: OR for each 10% increase in poverty = 0.82 (0.60, 1.13) although the raw data suggests the relationship may be non-linear. These differences disappeared in analyses adjusted for store type and other sociodemographic factors but CIs were wide. Giovenco (2018) argues that for locality-based evaluations unadjusted analyses may be more appropriate as they describe actual differences in compliance across low and high SES areas.<br>*Comments:* Data not presented on menthol sales prior to ban	possibly negative
Fry (2017)	Cross-sectional audit of compliance with a tobacco retailer notification (TRN) scheme and in-store tobacco retailing regulations in New South Wales. In relation to the TRN, overall one unlisted tobacco retailer was identified for every 12.6 that were listed. Both unadjusted and adjusted analyses found no significant difference in compliance by area-based SES although CIs were wide and results were almost significant for the highest vs the lowest SES areas in unadjusted analyses: OR = 2.16 (0.99, 4.72); p(trend)=0.094. In relation to in-store retailing regulations, overall 26.6% of audited retailers failed to comply with one or more of these regulations.	negative

	Levels of non-compliance were higher among retailers that were not registered with the TRN scheme: unadjusted OR = 2.24 (1.53, 3.28). Retailers located in high SES areas were less likely to be non-compliant: unadjusted OR for quintile 2 vs 1 (the most disadvantaged) = 0.83 (0.46, 1.50); 3 vs 1 = 0.73 (0.40, 1.35); 4 vs 1 = 0.55 (0.35, 0.88); 5 vs 1 = 0.68 (0.41, 1.16); p(overall) = 0.084; p(trend) = 0.043. Adjusted analyses gave a similar pattern of results with an overall p-value of 0.040 and a p-value for trend of 0.020.<br>*Comments:* Data not presented on in-store advertising and labelling practices prior to regulation	
Gibson (2015)	Experiment among current smokers in the USA comparing an FDA-proposed set of pictorial warning labels with existing text warnings across eight different outcomes. Overall, pictorial WLs were more effective than text WLs for the four outcomes most proximal to the warning (i.e. negative emotions plus intentions to talk about the warning, to engage in avoidance behaviours (which Gibson argues can increase the likelihood of quit-related activities), and to hold back from smoking). No overall differences were found in relation to perceived effectiveness and quit intentions. Analyses comparing low education smokers with the general population sample found no significant differences, leading the authors to conclude that pictorial WLs were unlikely to increase inequalities. While CIs were not supplied, parameter estimates suggest that some analyses may be underpowered (e.g. among low education smokers aged 18-25, pictorial WLs were more likely to elicit negative emotions than text WLs (β = 0.15, p<0.01) but no such effect was seen among the general population (β = 0.05, p=ns)).	mixed/unclear → lack of power
Giovenco (2018)	Empirical modelling study of the likely impact of a forthcoming ban on tobacco sales by pharmacies in New York City, looking at expected changes in tobacco retailer density as a result of the ban. On average, retailer density was expected to fall by approximately 6.8% across all areas (range = 0-50%), with larger decreases in more affluent/educated neighbourhoods and a minimal impact in poorer, less educated areas. Unadjusted analyses (which the authors argue are the most appropriate) suggested a negative equity impact on the percentage reduction in retailer density across two area-based SES measures: for each $10,000 increase in median income, the reduction in retailer density increased by 1% (0.68,1.32%); for each 10% increase in the proportion of adults with less than high school education, the reduction in retailer density decreased by 2.6% (1.84,3.34%). These results appear to be driven by a greater proportion of pharmacy-based tobacco retailers in higher income areas of NYC.	negative
Hummel (2015)	Analysis of policy triggers (warning labels) for thinking about quitting among current smokers in six European countries (France, Germany, the Netherlands, Ireland, Scotland and the rest of the UK), using multiple waves of the ITC Europe (national-level) longitudinal surveys. Overall, between 17-45% of respondents mentioned that warning labels had triggered thoughts of quitting. There was broadly consistent evidence of an equity positive (or possibly positive) effect, with low income and low education smokers being more likely to report warning labels as a trigger than high SES smokers across all six countries.<br>*Comments:* Focuses on current smokers only – those who quit successfully as a result of warning labels were excluded. Unclear whether outcome measure robust – retrospective, self-report that policy triggered thoughts of quitting in last 6 months	positive

Kuipers (2017)	Pre/post evaluation of a partial PoS display ban for large shops in England, using repeat cross-sectional surveys covering the general adult population. Overall, in fully adjusted models, there was a (non-significant) step-change decrease in smoking prevalence immediately following the ban (% change = -3.69 (-7.94, 0.75), p = 0.102), combined with a steepening of the existing downward trend (additional % annual change = -0.46 (-0.72, -0.20), p = 0.001). The pattern of change was somewhat different across occupational groups. Among manual workers (C2DE), although there did not appear to be an immediate large, step-change in prevalence (-1.82 (-6.91, 3.55), p = 0.500), there was a steeper decline in the downward trend (-0.62 (-0.93, -0.30), p<0.001) than that seen overall. Among non-manual workers (ABC1), there was large (marginally significant) step-change (-8.05 (-15.8, 0.40), p = 0.061) and a smaller (non-significant) increase in the downward trend (-0.42 (-0.90, 0.06), p = 0.084). Focusing only on the statistically significant results, the authors conclude that there is some evidence of stronger effect in low SES groups i.e. of an equity positive effect. Wide CIs make it difficult to be certain, however, about the lack of impact in high SES groups – the findings do not completely preclude the possibility of an immediate drop in prevalence followed by a more modest steepening of the downward trend. The combined impact of the step-change and trend-change were not explored.<br>*Comments:* Analysis of cigarette consumption also presented but showed no intervention effect either overall or by SES	mixed/unclear → step-change = possibly negative<br>trend = positive<br>overall effect = not assessed
Lee (2015)	Cross-sectional audit of compliance with FDA tobacco advertising and labelling regulations (including bans on self-service displays, vending machines and sales of single cigarettes) in the USA. Overall, 718 (2.1%) warning letters were issued from approximately 33,500 inspections, with the most common form of violation relating to the use of self-service displays (553). In unadjusted analyses, ORs for each 10% increase in the proportion of adults living in poverty varied according to the type of violation: self-service displays = 0.97 (0.79, 096) – possible error as parameter estimate not within CI; sales of single cigarettes = 1.54 (1.31, 1.81); presence of vending machines = 1.16 (0.90, 1.51); product mislabelling = 1.11 (0.83, 1.47). These differences disappeared when adjusting for the percentage of black/Latino inhabitants.<br>*Comments:* Data not presented on in-store advertising and labelling practices prior to regulation	mixed/unclear → self-service displays = mixed/unclear (possible error in results)<br>other regulations = negative
Moodie (2013)	Crossover RCT in a naturalistic setting among young adult (18-35 years old) female smokers in Scotland, comparing plain packaging and the participants' own brand packaging with respect to a range of intermediate outcomes. Overall, own brand packs were viewed more positively than plain packs in relation to feelings about smoking, pack perceptions and pack feelings. Differences in health warning salience and credibility were minimal but participants did report higher levels of warning attention and processing for the plain packs. Avoidant and cessation-related behaviours were more common with plain packs whereas cigarette consumption was lower. This pattern of results was broadly consistent across social grade, leading the authors to conclude that plain packaging might equally benefit both low and high SES groups, although they did express some caution in reaching this interpretation due to the relatively small sample size. As parameter estimates and CIs were not supplied, it was not possible to independently assess the statistical power of these analyses.	mixed/unclear → lack of power

Nagelhout (2016)	Quasi-experimental study across four EU countries comparing newly-implemented pictorial warning labels with existing text warnings, including both a pre/post and an intervention/control analysis, using data on current smokers from two waves of a longitudinal survey. Overall, there was a marginally significant increase in warning salience (noticing WLs) in one intervention country (UK) but decreases in both the other intervention country (France) and the two controls (Germany & Netherlands). A similar pattern was observed for cognitive responses (labels led to thinking) and forgoing cigarettes as a result of the WLs, whereas warning label avoidance increased across all four countries. Tests of interactions found that avoidance was higher among low/moderate educated smokers in the UK (OR = 2.25/3.21, p<0.001) and higher among high educated smokers in the Netherlands (OR = 3.52, p=0.036). No other differences by educational level were found, leading the authors to conclude that pictorial WLs had a neutral equity impact among continuing smokers. As parameter estimates and CIs were not supplied for the non-significant interactions, it was not possible to assess the statistical power of these analyses.<br>*Comments:* Focuses on current smokers only – participants who quit between waves were excluded even though exposed to WLs	mixed/unclear → unable to assess power of ns results; complex pattern
Swayampakala (2018)	Post-implementation evaluation (using data from multiple waves of a longitudinal survey with replacement of quitters) of "wear out" effects among current smokers following the introduction of updated, larger pictorial warning labels along with pack inserts (Canada) and plain packaging (Australia). Overall, in fully adjusted analyses, there was significant decline across both countries in WL attention over the two years following implementation (β = −0.02 (−0.038, −0.001)) but a significant increase in cognitive responses (β = 0.05 (0.02, 0.78)) and in forgoing of cigarettes (OR = 1.07 (1.03, 1.12)). There was some variation across SES groups. WL attention remained broadly stable in high income smokers but declined in low income smokers (p < 0.05), with a similar pattern across both countries. In contrast, cognitive responses increased among high SES smokers but remained broadly stable among low SES smokers – education: p(Ca, high vs low) = 0.012; p(Aus, high vs low) = 0.0242; income: p(Ca, mid vs low) < 0.05; p(Ca, high vs low) < 0.001; p(Aus, high vs low) < 0.05. No significant SES differences were found in relation to forgoing cigarettes. As parameter estimates and CIs were not supplied for the non-significant interactions, it was not possible to assess the statistical power of these analyses.<br>*Comments:* Immediate impact of the new WLs could not be assessed as outcome data was not presented for the existing, smaller warnings. Focuses on current smokers only – participants who quit between waves were excluded even though exposed to WLs. Based on online consumer panels so findings may not be generalisable	mixed/unclear → attention/ cognitive = negative<br>forgoing = mixed/ unclear (unable to assess power of ns results)
Thrasher (2015)	Post-implementation evaluation (using data from multiple waves of a longitudinal survey with replacement of quitters) of enhanced pack inserts in Canada, where the new inserts displayed pictorial warning labels (in contrast to the previous text warnings) emphasising the benefits of quitting and containing cessation advice. Overall, the proportion of current smokers who reported reading the inserts was around 26-31% across the five survey waves. Smokers with higher income levels were more likely to read the inserts: adjusted OR for income of $30-60k vs <$30k = 1.24 (1.01, 1.52); for income ≥$60k vs <$30k = 1.26 (1.03, 1.54). The pattern in relation to education was less clear cut. Compared to those with the lowest level of education (high school or less),	mixed/unclear → varies by SES

	smokers with a college education were less likely to read the inserts (AOR = 0.75 (0.63, 0.90)). While the equivalent estimates for those with a university degree were non-significant in the adjusted analysis (AOR = 1.11 (0.88, 1.41)), the relatively wide CIs do not allow us to rule out an effect.<br>*Comments:* Does not shed light on whether the new inserts were more effective than the existing ones in engaging smokers. Focuses on current smokers only – participants who quit between waves were excluded even though exposed to inserts. Based on online consumer panels so findings may not be generalisable	
Thrasher (2018)	Discrete choice experiment among current smokers in the USA to assess the extent to which pack inserts are motivating and helpful for smoking cessation, exploring five different sets of insert characteristics. Overall, inserts that contained imagery were perceived as being more motivating and more helpful than text-only inserts, as were inserts that contained cessation resource information. These positive effects were apparent across both low (less than degree) and high (degree or above) education groups although the direction of equity impact was different for the two outcomes. Tests of interaction found that the impact of image-based inserts was greater among high education smokers for both motivation ($p<0.001$) and helpfulness ($p<0.001$) although the difference in effect size was relatively small (for motivation: β(high) = 0.11, β(low) = 0.09; for helpfulness: parameter estimates not supplied). In contrast, the presence of cessation resource information was more motivating ($p<0.001$) and helpful ($p<0.001$) among low education smokers (for motivation: β(high) = 0.05, β(low) = 0.09; for helpfulness: β(high) = 0.05, β(low) = 0.08). The authors suggest that the unexpected negative equity effect for imagery might relate to the small proportion of low education smokers with an attainment level of high school or less (23% compared to 30% with some university or college education). Further analyses showed that the inclusion of testimonials and calls to action on the pack inserts had no impact on motivation and helpfulness, either overall or across educational groups.	mixed/unclear → imagery = negative cessation information = positive
Tucker-Seeley (2016)	Empirical modelling study of the likely impact on tobacco retailer density of a forthcoming, voluntary ban by CVS pharmacies on selling tobacco products in Rhode Island, together with an exploration of the potential impact of extending this ban to all pharmacies across the state. At baseline, prior to the ban, unadjusted analyses indicated a significant association with three area-based SES measures: retailer density decreased with increasing levels of income (β = -0.198 (-0.222, -0.173)) and education (β = -0.038 (-0.043, -0.033)) but increased with increasing levels of poverty (β = 0.033 (0.028, 0.039)). Parameter estimates were very similar in analyses excluding CVS pharmacies and all pharmacies, leading the authors to conclude that pharmacy bans on tobacco sales would have limited impact on inequalities in retailer density although the absence of an explicit analysis of change at tract-level makes it difficult to be certain. Tucker-Seeley suggests that this lack of effect may be due to the relatively small proportion of pharmacy-based tobacco retailers (CVS = 4.5%, all pharmacies = 10.0%) but, as the equivalent figure for all pharmacies in the Giovenco study was 6.2%, this is unlikely to be the entire explanation. Contextual factors are also likely to play a part since retailer density at baseline was seen to increase with increasing SES in NYC (Giovenco) but decrease with increasing SES in Rhode Island (Tucker-Seeley).<br>*Comments:* Lack of formal statistical analysis of change at tract-level	neutral

Smith CE, *et al. Tob Control* 2021; 30:e87–e95. doi: 10.1136/tobaccocontrol-2020-055874

Zacher (2014)	Repeat cross-sectional observational study examining changes in active smoking, pack display and pack concealment among patrons of outdoor venues following the introduction of plain packaging with updated, larger pictorial health warnings in South Australia (Melbourne and Adelaide). Zacher argues that reductions in pack display and increases in concealment are positive outcomes since they result in less exposure to indirect advertising and smoking cues. Overall, the incidence rate ratio (IRR) of active smoking among patrons declined by 23% and of pack display by 15% (largely due to the reduction in active smokers). These reductions were apparent in mid and high-SES areas but not in low SES areas (test of interaction for active smoking: p<0.001; pack display: p=0.045), suggesting a negative equity impact on these outcomes. Parameter estimates were provided for pack display only: IRR for low SES = 0.96 (0.84, 1.09); mid = 0.85 (0.76, 0.96); high = 0.76 (0.68, 0.86). Across all areas, the IRRs for pack concealment and cigarette case use increased with the introduction of the new packaging whereas the IRR for face-up pack placement decreased. SES interactions were significant for concealment (p<0.001) and face-up placement (p=0.047), with the highest rates of concealment and the lowest rates of face-up placement in low-SES areas, suggesting a positive equity impact for both outcomes. IRRs for concealment: low SES = 4.93 (2.47, 9.85); mid = 1.59 (0.93, 2.72); high = 1.88 (1.07, 3.29). IRRs for face-up pack placement: low SES = 0.70 (0.57, 0.84); mid = 0.88 (0.76, 1.02); high = 0.98 (0.84, 1.14). Interactions for case use were non-significant but parameter estimates and CIs were not provided, preventing an assessment of statistical power.	mixed/unclear → smoking/ pack display = negative<br>pack concealment/ face-up = positive<br>case use = mixed/ unclear (not able to assess power of ns results)
Population-level cessation support interventions (16 studies)		
Benson (2016)	Evaluation of the impact of a national reimbursement scheme for cessation medications in the Netherlands during 2011 on the proportion of quit attempts using pharmacotherapy, drawing on data from a continuous cross-sectional survey (national-level) between 2009-2012. Stratified analyses (with CIs) were presented by SES (income/education) and by year but formal tests of interaction between intervention effectiveness and SES were not reported. In relation to education, there was some evidence from MV analyses (adjusted for injunctive norms) that the odds of using pharmacotherapy in a quit attempt increased in 2011 compared to the other study years (2009-10 & 2012) among both middle (OR=1.29 (0.71, 2.36)) and high (OR=1.75 (0.92, 3.35)) educated smokers – but decreased among the low education group (0.89 (0.39, 2.02)). In contrast, the reverse pattern was seen in relation to income, with a higher odds of an increase in pharmacotherapy use among low income smokers (OR=1.54 (0.74, 3.21)) than among middle (OR=1.20 (0.50, 2.86)) and high (OR=1.21 (0.57, 2.58)) income smokers. None of these differences reach statistical significance.<br>*Comments:* Amalgamation of 2009/10 and 2012 for the non-intervention years might mask any residual effects on pharmacotherapy use after the reimbursement scheme had been disbanded. Relatively high levels of missing data on income (approximately a quarter of participants) – with over-representation of low-SES smokers (by education) among those without such data – may limit generalisability of income-based analyses	mixed/unclear → varies by SES
Bosdreisz (2016)	EU-wide analysis of the association between cessation support (TCS domain score) and the quit ratio/ smoking intensity across 27 member states, combining 3 waves of an ongoing cross-sectional survey with published TCS	mixed/unclear → quit ratio = negative

Smith CE, *et al. Tob Control* 2021; 30:e87–e95. doi: 10.1136/tobaccocontrol-2020-055874

	data. Overall, no significant associations were found with cessation for either outcome (<u>quit ratio</u>: OR=1.02 (0.99, 1.05); <u>intensity</u>: β=0.03 (-0.12, 0.18)). Analyses of interactions between cessation support and education indicated an equity negative effect in relation to the quit ratio (OR(high vs low)=1.08 (1.05, 1.11)). Differences in relation to smoking intensity were minimal indicating an equity neutral effect – although relatively wide CIs suggest the need for caution (β(high vs low)=-0.03 (-0.19, 0.14)).<br>*Comments:* Cross-sectional analysis makes it difficult to establish a causal relationship between TCS domain score and the quit ratio/smoking intensity	intensity = neutral
Campbell (2014)	Analysis of socioeconomic differences in quitline use across 7 unidentified provinces in Canada, using service monitoring data combined with national survey estimates of provincial smoking prevalence rates. Reach ratios for the lowest education group were greater than 1 in all 7 provinces (suggesting an overall equity positive effect) – with results being statistically significant in 5 provinces, marginally significant in province F (ReRa=1.17 (0.99, 1.37)), and unclear in province G (ReRA=1.07 (0.91, 1.25)). The largest effect was seen in province B (ReRa=1.77 (1.46, 2.16)).<br>*Comments:* Restricted to 7 provinces (unclear how selected) plus relatively high levels of missing data on education (19%) so findings may not be generalisable. Possible inconsistencies in data definitions across difference provinces	positive
Clare (2014)	Cross-sectional analysis of socioeconomic differences in use of telephone quit-lines and prescribed cessation medication among current smokers and recent quitters in Australia, using data from 4 consecutive waves an ongoing survey (national-level) between 2001-10. Overall, use of both forms of support was low (quitline=3.2%; prescribed medication=5.7%). Equity impact findings for quit-line use were mixed, with a significant negative effect for education (OR(university vs high school)=1.22 (0.91, 1.64), p=0.023) but borderline positive effects for income (OR(top 2/3 vs bottom 1/3)=0.81 (0.64, 1.02), p=0.073) and SEIFA (OR(highest vs lowest)=0.71 (0.52, 0.96), p=0.065). In contrast, use of prescription cessation medication suggested a positive equity effect across all three SES measures (<u>education:</u> OR(high vs low)=0.72 (0.56, 0.93), p=0.009; <u>income:</u> OR(high vs low)=0.93 (0.77, 1.11), p=0.426; <u>SEIFA:</u> OR(high vs low)=0.58 (0.46, 0.74), p<0.001)). In relation to recent quitting, an analysis of the interaction between education and prescribed medication use showed an equity positive effect (p(interaction)=0.005), such that medication use increased the odds of quitting among those with a high school education or less (OR(use vs no use)=2.50 (1.94, 3.23) but had no effect among those with a university degree (<u>no use:</u> OR(university vs high school)=1.98 (1.74, 2.24); <u>use:</u> OR(university vs high school)=1.94 (0.75, 5.05)). No other interactions were reported.<br><u>Comments:</u> Some uncertainty around which support types constitute a population-level intervention as precise definitions were not provided – data were also presented on asking doctor for help/reading quit literature/using other cessation mediation. Cross-sectional analysis makes it difficult to establish a causal relationship between medication use and recent quitting. Self-report measure of recent quitting may not be robust	mixed/unclear →<br>quitlines = mixed/ unclear (varies by SES)<br>medication = positive
Dhalwani (2014)	Analysis of socioeconomic differences in GP prescribing of NRT to pregnant smokers aged 15-49 in the UK, using routinely collected GP-practice data from the THIN database between 2006-12. Overall, NRT was	positive

	prescribed to approximately 11% of pregnant smokers. There was a significant association between Townsend deprivation score and NRT prescribing (p<0.001), with higher prescription rates among the most disadvantaged smokers (OR(most vs least)=1.29 (1.15, 1.45)), indicating an equity positive effect. *Comments:* Analysis does not adjust for level of nicotine dependence or for frequency of consulting GP. Focuses only on NRT and does not examine prescribing patterns for other (potentially more effective) cessation medications. Pre/post data not provided by SES so unable to explore equity impact of change in prescribing rules to cover pregnant smokers	
Douglas (2013)	Analysis of socioeconomic differences in the extent to which GPs offer cessation advice and prescribe cessation medications (NRT, bupropion and/or varenicline) to adult current smokers in the UK, using routinely-collected GP-practice data from the THIN database between 2008-10. Overall, 53.1% of smokers received cessation advice from their GP over the study period and 16.5% received at least one prescription for a cessation medication. There was evidence of an equity positive effect in relation to both interventions, with the odds of receiving advice increasing with increasing deprivation (Townsend: OR(most vs least)=1.28 (1.19-1.37); Mosaic: OR(F(people living in social housing in deprived areas) vs A (successful professionals living in desirable areas))=1.35 (1.20, 1.52)) and the odds of receiving a prescription also increasing with increasing deprivation (Townsend: OR(most vs least)=1.16 (1.05-1.28); Mosaic: OR(G(low-income families living in estate-based special housing) vs A (successful professionals living in desirable areas))=1.50 (1.31, 1.73)). *Comments:* Analysis does not adjust for level of nicotine dependence or for frequency of consulting GP. Validity of outcome measure (cessation advice) unclear - Read codes not provided; definition not fixed and may vary across GPs and between GPs/patients; possible under-recorded if multiple topics covered in consultation	positive
Hamilton (2016)	Pre/post evaluation of a local version of a national financial incentives scheme (QOF+) for GPs in the London borough of Hammersmith and Fulham (UK), assessing the impact of the scheme on cessation advice and/or SSS referral by GPs, using routinely collected data from electronic patient records. Overall, the proportion of male smokers receiving cessation advice/referral increased from 32.7% to 54.0% after the introduction of QOF+, with broadly similar increases across IMD levels (least deprived: OR(post vs pre)=2.20 (1.81, 2.66); most deprived: OR(post vs pre)=2.32 (2.00, 2.69)), suggesting an equity neutral effect. Among females, the proportion receiving advice increased from 35.4% to 54.1% although here there appeared to be a smaller improvement in the most deprived smokers ((least deprived: OR(post vs pre)=2.25 (1.83, 2.76); most deprived: OR(post vs pre)=1.89 (1.59, 2.25)). *Comments:* Greater ethnic diversity among local population suggests may not generalise to wider UK. Validity of outcome measure (cessation advice) unclear - Read codes not provided; definition not fixed and may vary across GPs and between GPs/patients; possible under-recorded if multiple topics covered in consultation	mixed/unclear → males = neutral females = possibly negative
Hardy (2014)	Analysis of socioeconomic differences in the extent to which GPs offer cessation advice to pregnant smokers aged 15-49 in the UK, using routinely collected GP-practice data from the THIN database between 2006-09. Overall, approximately 26-29% of pregnant smokers had cessation advice recorded in their notes over the study period. There was a significant association between Townsend deprivation score and recorded cessation advice	positive

Smith CE, *et al. Tob Control* 2021; 30:e87–e95. doi: 10.1136/tobaccocontrol-2020-055874

	(p<0.001), with smokers from the most deprived areas being more likely to receive advice than smokers from the least deprived areas (OR=1.38 (1.14, 1.68), suggesting an equity positive effect.<br>*Comments:* Validity of outcome measure (cessation advice) unclear - Read codes not provided; definition not fixed and may vary across GPs and between GPs/patients; possible under-recorded if multiple topics covered in consultation	
Hummel (2015)	Analysis of policy triggers (availability of free or low-cost cessation medication) for thinking about quitting among current smokers in five European countries (Germany, the Netherlands, Ireland, Scotland and the rest of the UK), using multiple waves of the ITC Europe (national-level) longitudinal surveys. Overall, between 18-48% of respondents mentioned that the availability of free or low-cost cessation medications had triggered thoughts of quitting. There was broadly consistent evidence of an equity positive (or possibly positive) effect, with low income and low education smokers being more likely to report free/low-cost medication as a trigger than high SES smokers across all five countries.<br>Comments: Focuses on current smokers only – those who quit successfully as a result of the availability of free/low-cost cessation medication were excluded. Unclear whether outcome measure robust – retrospective, self-report that policy triggered thoughts of quitting in last 6 months	positive
Molarius (2017)	Cross-sectional analysis of socioeconomic differences in the receipt of smoking cessation advice during a primary care consultation among adults in 4 Swedish counties, using data from a population survey (study-specific) combined with demographic data from the national registry. Overall, 6.2% of adults (including non-smokers) who visited a primary care centre in the past 3 months received smoking cessation advice during their most recent consultation. In multivariate analyses adjusted for smoking status, respondents with the lowest level of education (compulsory only) were more likely to have received advice than those with post-secondary education (OR=1.6 (1.3, 2.1)), indicating an equity positive effect.<br>*Comments:* Restricted to respondents from 4 counties who visited a primary care centre in last 3 months – plus response rate of 51% with over-representation of younger, more educated adults - so findings may not be generalisable	positive
Nagelhout (2014)	Pre/post evaluation of the impact of a national reimbursement scheme for cessation medications in the Netherlands during 2011 on a range of outcomes measures (awareness of reimbursement possibilities, awareness of media activity, quit attempts, use of cessation support and quit success), drawing on data from three waves of a longitudinal survey (ITC national-level) between 2010-2012. Overall, following the introduction of the scheme, there was significant increase in quit attempts (OR=2.02, p<0.001) and quit success (OR=1.47, p<0.001) but levels of support use remained unchanged. Awareness of reimbursement possibilities also increased from 11% to 42% (OR=6.38%, p<0.001) but only awareness of the linked media campaign was associated with an increase in quit attempts (OR=1.95, p<0.001). Interactions between SES (household income) and survey wave for all outcome measures were presented but none were statistically significant at the 0.004 level (threshold adjusted to reflect multiple comparisons). Stratified analyses were not presented, making it	mixed/unclear → unable to assess power for ns results

	difficult to draw any firm conclusions about equity impact, particularly as ORs comparing low to high SES groups were not always close to 1 and CIs were typically wide.<br>*Comments:* Respondents who were lost to follow-up were younger, more educated and more likely to be female – plus income data was not available in more than a quarter of cases – so findings might not be generalisable. Absence of control group makes it difficult to draw causal inferences in relation to overall effect. Robustness of self-report measure of quit success unclear	
Sadasivam (2013)	Evaluation of a web-assisted tobacco intervention (Decide2Quit) that recruited smokers in the USA via medical and dental practices, as well as via Google advertisements, drawing on data from the intervention registration database together with population comparison data from an ongoing cross-sectional survey (national-level). A greater proportion (p=0.001) of website-registered smokers had at least some college education (65.7%) than smokers in the general population (45.9%), indicating an equity negative effect. Smokers recruited through medical practices were, however, less likely to be college graduates (15.7%) – the same proportion as in the general population – than those recruited through dental practices (28.4%) and google (32.0%), suggesting that the additional referral pathways may have reduced, but not eradicated, inequalities in recruitment. Non-college graduates also made less use of the website than college graduates, with an incidence rate ratio of 0.7 (0.6, 0.9).<br>*Comments:* Lack of comparative educational categories for overall and source-specific analysis of recruitment makes it difficult to judge how much extended referral pathways may have reduced inequalities. No population demographic data provided for the areas covered by the participating practices (medical=81; dental=51) – not clear whether they representative of the wider population and whether the findings are likely to generalise if scaled to include all practices	negative (but possibly reducing)
Schauer (2014)	Cross-sectional analysis of socioeconomic differences in quit-line awareness and utilisation among adults (non-smokers and smokers) in the USA, drawing on data from a single wave of an ongoing national survey combined with estimates of state-level TC spending per smoker. Overall, 33.9% of respondents were aware of the quit-line and 7.8% of quit-line aware smokers who had made an quit attempt in the past year had made use of the quit-line. In unadjusted analyses, levels of awareness increased with increasing education and increasing income among all respondents regardless of smoking status (current, former and never). Similarly, in a MV adjusted analysis focused on current smokers who had made a (unsuccessful) quit attempt in the past year, awareness was lower among those with less than a high school education (OR(vs >high school)=0.73 (0.55, 0.98)) and among those with an income under $30k (OR(vs ≥$50k)=0.69 (0.56, 0.86)), indicating an equity negative effect. Equity impact findings were less clear cut for quit utilisation among quit-line aware smokers (current and former) who had made a quit in the last year, with possibly higher use among those with less than a high school education (OR(vs >high school)=1.14 (0.65, 2.00)) but a non-linear pattern by income (OR(<$30k vs ≥$50k)=0.96 (0.57, 1.61); OR($30-50k vs ≥$50k)=1.56 (0.94, 2.61)).<br>*Comments:* Robustness of retrospective, self-report measure of quit-line utilisation unclear	mixed/unclear → awareness = negative use = mixed/unclear (varies by SES)

Smith CE, *et al. Tob Control* 2021; 30:e87–e95. doi: 10.1136/tobaccocontrol-2020-055874

Skinner (2017)	Analysis of socioeconomic differences in the receipt of subsidised cessation medications by older adult (≥ 45 years) smokers In Australia, using baseline survey data from a wider cohort study, combined with data on subsequent prescriptions from two national benefits scheme. Overall, 34.1% of smokers were supplied with at least one cessation medicine by the end of the follow-up period, with 29% receiving varenicline, 6.83% NRT and 2.02% bupropion. In relation to education, there was some evidence of an equity positive effect for all three medications such that university graduates had a lower odds of receiving treatment than those with no school certificate – although this difference did not reach statistical significance for bupropion (OR(varenicline=0.79 (0.70, 0.90); OR(NRT)=0.77 (0.60, 0.99); OR(bupropion)=0.79 (0.52, 1.19)). In relation to area-based deprivation, there appeared to be a non-linear relationship for both varenicline and NRT, with a higher odds of receiving treatment in the mid-deprivation quintiles than in the most/least disadvantaged areas – although for varenicline the difference between the most and least advantaged areas was still significant (OR=1.16 (1.02, 1.32)). In contrast, ORs for bupropion were closer to 1 (ranging from 0.98 to 1.09) although wide CIs and the lack of a clear trend meant conclusions could not be drawn regarding equity impact.<br>*Comments:* Sample may not be representative (e.g. over-sampling of smokers ≥ 80 years old and from non-urban areas)	mixed/unclear → complex pattern that varies by SES
Taylor (2017)	Analysis of the relative effectiveness of varenicline vs NRT in promoting long-term smoking cessation within a primary care setting, using a prospective cohort design based on GP-practice data from the CPRD database including smokers first treated between 2006-15 – and followed-up at 3/6/9 months as well as 1/2/4 years. Overall, 32.1% of participants were prescribed varenicline, and the odds of being quit were higher for those receiving varenicline than for those receiving NRT at all follow-up points (e.g. 2-year OR=1.26 (1.23, 1.29)). Participants living in higher deprivation areas (IMD rank 11-20) were less likely to be prescribed varenicline than those living in the lower deprivation areas (OR=0.91 (0.90, 0.92)), indicating an equity negative effect. In relation to quit outcomes, there was some evidence that during the first year of follow-up varenicline might be slightly less effective in more disadvantaged rather than less disadvantaged smokers but differences were small and non-significant (e.g. higher deprivation at 3 months: OR(varenicline vs NRT)=1.38 (1.31, 1.46); lower deprivation at 3 months: OR(varenicline vs NRT)=1.45 (1.37, 1.54)). By the second and fourth year of follow-up, this difference had all but disappeared (e.g. higher deprivation at 2 years: OR(varenicline vs NRT)=1.28 (1.23, 1.34); lower deprivation at 2 years: OR(varenicline vs NRT)=1.29 (1.23, 1.35)), suggesting an equity neutral effect on quit outcomes in the longer term.	negative overall → reach = negative success (3 months) = possibly negative success (2 years) = neutral
Vaz (2017)	Evaluation of a national network of NHS stop smoking services for pregnant women (SSSP) in the UK, combining a cross-sectional survey (study-specific) with an analysis of published data from 3 sources – SSSP performance monitoring data, hospital episode statistics and the 2011 UK census. Overall, mean SSSP reach (% pregnant smokers setting quit date with SSSP) was 17.2% and mean effectiveness (% SSSP quitters who abstinent at 4 weeks) was 45.2%. In the bivariate response model, there was evidence of an equity positive effect in relation to reach, with a 0.55% increase (0.25%, 0.85%) in reach for every unit increase in IMD, and a 0.31% decrease (-0.59%, -0.03%) for every unit increase in % of people residing in SSSP area who are in	mixed/unclear → reach = positive effectiveness = negative overall effect = not assessed

	managerial or professional occupations. In contrast, there was evidence of an equity negative effect in relation to SSSP effectiveness, with a 0.51% decrease (-0.95%, -0.07%) in effectiveness for every unit increase in % of people (≥ 16) residing in SSSP area who have no qualifications. Estimates were not provided for the combined equity impact of reach and effectiveness at the population-level across each of the three SES measures. *Comments:* Based on small-area census data so not clear to what extent findings will apply to individual SES. Does not include estimates of combined equity impact across reach and effectiveness	
Other interventions (2 studies)		
Hu (2016)	Evaluation of the English Government strategy to tackle health inequalities (1999-2010), using a modified difference-in-difference analysis to assess changes in smoking inequality trends both pre/post implementation and compared to 3 European control countries, using data from three corresponding waves of an ongoing cross-sectional survey (national-level) within each country. While the decline in smoking prevalence in England was steeper among both high and low educated respondents in 2000-2010 (post-implementation) compared to 1990-2000 (pre-implementation), the decline was greater among high educated respondents, resulting in a (non-significant) worsening of smoking inequalities post-implementation (OR(change in trend)=1.19; SE=0.182). Relative to the control countries, this widening in inequalities appeared to be slightly smaller than that observed in Finland (OR(E vs F)=0.93; SE=0.267) but larger than that observed in the Netherlands (OR(E vs N)=1.20; SE=0.270) and Italy (OR(E vs I)=1.23; SE=0.209).	possibly negative
Stafford (2014)	Evaluation (with matched controls and SES-stratified national comparators) of a 10-year, area-based initiative (New Deal for Communities) targeted at areas of high deprivation in England between 1998-2007 with the aim of reducing socioeconomic inequalities in health, combining data from multiple waves of a study-specific panel survey (2002, 2004, 2006, 2008) with data from corresponding years of an ongoing cross-sectional, national (HSE) survey. Among all study participants (including NDC intervention/comparator areas as well as respondents to the national survey), there was a statistically significant decline in smoking prevalence between 2002-2008 (OR(2-year)=0.95 (0.93, 0.97)). Compared to low deprivation HSE respondents, the (relative) rate of decline was less steep in NDC intervention areas (OR=1.03 (1.01, 1.06)), in NDC comparator areas (OR=1.04 (1.01, 1.07)), and in high deprivation HSE respondents (OR=1.05 (1.02, 1.08)). *Comments:* Data on smoking prevalence not presented immediately prior to, nor during the early years of, the NDC intervention so unable to assess whether intervention equity impact has changed. Relies on the amalgamation of surveys with different sampling frames, data collection instruments and analytical strategies which may limit the comparability of NDC and HSE respondents	mixed/unclear → complex pattern
Multiple policies (5 studies)		
Bosdriesz (2015)	Repeat cross-sectional, national survey of the relationship between smoking cessation/intensity and the TCS over a 24-year period in the Netherlands, with a separate analysis for 1988-00 (a time when the level of TC	mixed/unclear →

	activity was relatively low and stable) and 2001-11 (a time of more intense and evolving activity). Overall, during the first study period, there was a non-significant, negative association between the quit ratio and TCS in the concurrent and previous three years (e.g. OR(concurrent year)=0.95 (0.89,1.01)). In contrast, during the second study period, there was a significant and positive association between the quit ratio and TCS in the previous two years (OR(1-year lag)=1.15 (1.05, 1.26), OR(2-year lag)=1.20 (1.10, 1.30)). While ORs were virtually identical across low- and high-education groups during 1988-00, TCS x SES interactions were significant (p ranging from <0.001 to 0.050) at all lags during 2001-11 (although the individual ORs were only significant for the 1-year and 2-year lags). More specifically, stratified analyses suggested that the TCS effect may have occurred earlier in high- than low-education smokers, such that the largest OR was at 1 year for the high-SES group (OR(1-year lag)=1.24 (1.12, 1.37), OR(2-year lag)=1.17 (1.03, 1.32)) and at 2 years for the low-SES group (OR(1-year lag)=1.14 (1.02, 1.27), OR(2-year lag)=1.23 (1.12, 1.34)). The closeness of the individual estimates and the relatively wide CIs make it difficult to draw firm conclusions despite the significant interactions. In relation to smoking intensity, the overall association with TCS during 2001-11 (the only years for which data was available) was non-significant and negative at the 1-3 years lags (e.g. β(1-year lag)=-0.20 (-0.55, 0.15)). TCS x SES interactions were again significant at all lags (p ranging from 0.006 to 0.013), with parameter estimates very close to zero in the low-education group albeit with wide CIs (e.g. β(1-year lag)=-0.05 (-0.41, 0.31)) but with stronger negative associations in the high-SES group (e.g. β(1-year lag)=-0.62 (-1.19, -0.05)). Thus, mean smoking intensity appeared to decline with increasing TCS among high-educated but not among low-educated smokers, although the effect size was small (β=-1 corresponds to an average decrease of 1 cigarette per day for each 10% increase in TCS).<br>*Comments:* The quit ratio (no. former smokers /(no. former smokers + no. current smokers)) may be relatively insensitive to detecting short-term effects on cessation, possibly leading to the underestimation of any association with TCS	quit ratio = complex pattern<br>intensity = negative
Bosdreisz (2016)	EU-wide analysis of the association between TCS and the quit ratio/smoking intensity across 27 member states, combining 3 waves of an ongoing cross-sectional survey with published TCS data. Overall, no significant associations were found with TCS for either outcome (<u>quit ratio</u>: OR=1.02 (0.96, 1.08); <u>intensity</u>: β=-0.16 (-0.49, 0.17)). Analyses of interactions between TCS and SES indicate an equity negative effect in relation to education for both the quit ratio (OR(high vs low)=1.13 (1.08, 1.19)) and smoking intensity (β(high vs low)=-0.27 (-0.55, -0.01)). In contrast, differences in relation to occupational group were smaller and non-significant, suggesting an equity neutral effect (<u>quit ratio</u>: OR(non-manual vs manual)=1.02 (0.96, 1.07)); <u>intensity</u>: β(non-manual v manual)=0.12 (-0.14, 0.39)).<br>*Comments:* Cross-sectional analysis makes it difficult to establish a causal relationship between TCS and the quit ratio/smoking intensity	mixed/unclear → varies by SES
Havard (2018)	Pre/post evaluation of the impact on smoking during pregnancy of two different TC interventions introduced at the approximately same time (graphic warning labels on cigarette packs in March 2006 and an extended partial SF law phased in between July 2005-06) in New South Wales (Australia), using an interrupted time series	mixed/unclear → non-linear SES effect

	analysis (segmented regression) of birth records combined with hospital admissions data. Smoking during pregnancy fell from 17.1% in June 2003 to 10.6% in December 2011, representing a -0.39% relative monthly decrease in prevalence over the study period. Stratified analyses (with CIs) were presented by SES but differences by area deprivation were not formally tested. Across the whole study period, the rate of decline decreased with increasing deprivation (low=-0.43% (-0.48%, -0.37%); mid=-0.36% (-0.32%, -0.40%); high=-0.20% (-0.24%, -0.16%)). Overall, there was a non-significant dampening of the downward trend following the introduction of the two new policies (change in trend = +0.25% (-0.19%, +0.69%)). This attenuation of the trend was seen across all SES levels but was smaller in the mid-deprivation groups (low: change in trend = +1.29% (+0.41%, +2.17%); mid: change = +0.29% (-0.33%, +0.90); high: change = +0.59% (0.0%, +1.19%)).<br>*Comments:* Paper also looks at a number of other interventions not included in this review as they had no effect either overall or by SES (i.e. exposure to general anti-smoking TV adverts and a complete SF ban)	
Hu (2017)	Difference-in-difference analysis of the relationship between country-level non-price TC index (covering four different domains) across 9 European countries during 1990-2007, using 33 waves of national-level, cross-sectional health surveys. Overall, there was a significant decline in smoking prevalence with increasing non-price TC index among men in both the model adjusting for cheapest cigarette price (OR=0.95 (0.91, 0.99), and in the model adjusting for popular cigarette price (OR=0.96 (0.92, 0.995)). Parameter estimates were almost identical for women but did not reach statistical significance due to wider CIs. Analyses by education and occupation found minimal differences in the ORs among men for both SES measures and among women for occupation (all tests for SES x non-price index interaction were non-significant), suggesting a neutral equity effect. Differences in the ORs for education among women were larger, with the negative association only being seen in the low education group, although the test for interaction was still non-significant (low: OR=0.94 (0.85, 1.05); high: OR=1.00 (0.95, 1.05); p(interaction=0.444).<br>*Comments:* Mass media campaigns not covered by non-price TC index	mixed/unclear → males = neutral females = mixed/ unclear (varies by SES)
Pinilla (2017)	Repeat cross-sectional national survey in Spain, using retrospective data on quitting in the previous 5 years to explore SES patterns in cessation, with separate analyses for 2001-05 (a period of relatively low TC activity) and 2006-10 (following the introduction of Law 28 in 2006). Overall, the proportion of smokers quitting within 5 years increased from 12% in the 2001 cohort to 20% in the 2006 cohort, with a steady divergence in the proportion having quit over the 5-year period. Pre/post analyses by SES were not conducted but rather SES patterns were presented separately for each cohort. In relation to social class, smokers in the lowest SES group were less likely to quit than those in the highest SES group in both cohorts (2001 cohort: HR=0.76, SE=0.10, p<0.01; 2006 cohort: HR=0.67, SE=0.09, p<0.01). Interactions between cohort (i.e. intervention) and SES were not formally tested, making it difficult to come to any firm conclusions about intervention equity effect (particularly given the relatively large standard errors) although if anything inequalities appear to have widened. The pattern was somewhat different in relation to education. In the 2001 cohort, the highest SES smokers were again more likely to quit than the lowest SES smokers (HR=1.49, SE=0.16, p<0.01), but these inequalities appear to have largely	mixed/unclear → varies by SES

Smith CE, *et al. Tob Control* 2021; 30:e87–e95. doi: 10.1136/tobaccocontrol-2020-055874

disappeared in the 2006 cohort (HR=1.08, SE=0.15, p=ns). While this may be suggestive of a positive equity effect, the lack of formal a pre/post analysis, coupled with relatively wide standard errors, again makes it difficult to draw firm conclusions. Moreover, without data on the underlying quit rates, it is not clear whether this apparent reduction in inequalities stems from an improvement in the low education group or a decline in the high education group.
Comments: In the absence of a control and/or a more detailed time series analysis, findings may be explained by secular trends and/or concomitant policy initiatives. Reliance on retrospective reports may reduce reliability of quit history data

S4. Quality appraisal results

Equity focus:
2 = yes – on SES inequalities; 1 = yes – but not exclusively SES; 0 = no
[Studies that did not primarily focus on equity may be of high quality in relation to their main research question but be sub-optimal for the assessment of SES inequalities. Those examining a broader range of inequalities (e.g. gender, ethnicity) may be more optimally designed to evaluate equity impact but be unable to report the required level of detail in relation to SES.]
Generalisable:
1 = yes – at national or state/regional/local level; 0 = no
[Based on representativeness of study sample in relation to source population, and (as appropriate) response &/or attrition rates.]
Adequately powered SES analysis:
2: yes – for all analyses; 1: yes – for some analyses; 0: no or not possible to assess
[As formal power calculations were typically not provided, power was assessed via inspection of confidence intervals - with narrow CIs being required to support an equity neutral finding. Studies which did not report formal statistical analyses could not be assessed.]
Confounding addressed:
1 = yes – adjustment for at least one factor; 0 = none reported

	Equity focus	Generalisable	Adequately powered SES analysis	Confounding addressed	Value to review
Price/taxation increases (16)					
Bosdriesz (2016)	2	1	2	1	6
Choi (2013)	0	0	1	1	2
Choi (2018)	1	1	1	1	4
Goldin (2013)	2	1	2	1	6
Havard (2018)	1	1	1	1	4
Hawkins (2014)	1	0	2	1	4
Hu (2017)	2	1	0	1	4
Hummel (2015)	2	0	1	1	4
Keeler (2018)	1	1	0	1	3
MacLean (2016)	1	1	0	1	3
Mayne (2018)	2	0	1	1	4
Parks (2017)	2	1	2	1	6
Regidor (2015)	2	1	0	1	4
Vijayaraghavan (2013)	2	1	0	1	4
Yu (2018)	1	1	0	1	3
Zhang (2013)	2	1	2	1	6
Smoke-free policies (17)					
Babb (2017)	2	1	2	0	5
Berg (2015)	1	0	2	1	4
Bosdriesz (2016)	2	1	2	1	6
Carton (2016)	1	1	0	1	3
Farley (2015)	2	0	1	1	4

Smith CE, *et al. Tob Control* 2021; 30:e87–e95. doi: 10.1136/tobaccocontrol-2020-055874

Gentzke (2018)	2	0	1	1	4
Hood (2014)	1	1	2	1	5
Huang (2015)	2	1	1	1	5
Hummel (2015)	2	0	1	1	4
Lidon-Moyano (2017)	0	1	0	1	2
Murphy-Hoefer (2014)	1	1	0	0	2
Platter (2018)	0	0	0	0	0
Regidor (2015)	2	1	0	1	4
Sandoval (2018)	2	1	1	1	5
Schechter (2018)	1	0	0	0	1
Tchicaya (2016)	2	1	1	1	5
Zhang (2013)	2	1	1	1	5
Mass media campaigns (11)					
Bosdriesz (2016)	2	1	1	1	5
Durkin (2018)	2	1	1	1	5
Havard (2018)	1	1	0	1	3
Kim (2018)	2	0	2	1	5
Lewis (2015)	0	1	0	1	2
McAfee (2017)	1	1	1	1	4
Neff (2016)	0	1	0	1	2
Nonnemaker (2014)	2	1	0	1	4
Rayens (2016)	1	0	1	1	3
Vallone (2015)	1	1	1	1	4
Zhang (2013)	2	1	2	1	6
Controls of advertising, promotion and marketing of tobacco (15)					
Bosdriesz (2016)	2	1	1	1	5
Czaplicki (2018)	1	1	0	1	3
Fry (2017)	1	1	1	1	4
Gibson (2015)	2	0	0	1	3
Giovenco (2018)	1	1	2	0	4
Hummel (2015)	2	0	1	1	4
Kuipers (2017)	2	1	1	1	5
Lee (2015)	2	0	1	1	4
Moodie (2013)	0	0	0	1	1
Nagelhout (2016)	2	0	0	1	3
Swayampakala (2018)	1	0	1	1	3
Thrasher (2015)	0	0	1	1	2
Thrasher (2018)	1	0	2	1	4

Smith CE, *et al. Tob Control* 2021; 30:e87–e95. doi: 10.1136/tobaccocontrol-2020-055874

Tucker-Seeley (2016)	2	1	2	0	5
Zacher (2014)	0	0	1	1	2
Population-level cessation support interventions (16)					
Benson (2016)	2	1	0	1	4
Bosdriesz (2016)	2	1	2	1	6
Campbell (2014)	1	0	1	0	2
Clare (2014)	2	1	1	1	5
Dhalwani (2014)	1	1	2	1	5
Douglas (2013)	2	1	2	1	6
Hamilton (2016)	2	1	1	1	5
Hardy (2014)	1	1	2	1	5
Hummel (2015)	2	0	1	1	4
Molarius (2017)	2	0	2	1	5
Nagelhout (2014)	2	0	0	1	3
Sadasivam (2013)	0	1	2	0	3
Schauer (2014)	1	1	1	1	4
Skinner (2017)	2	0	1	1	4
Taylor (2017)	2	1	1	1	5
Vaz (2017)	0	1	2	1	4
Other interventions (2)					
Hu (2016)	2	1	0	1	4
Stafford (2014)	2	1	2	1	6
Multiple policies (5)					
Bosdriesz (2015)	2	1	2	1	6
Bosdriesz (2016)	2	1	2	1	6
Havard (2018)	1	1	0	1	3
Hu (2017)	2	1	1	1	5
Pinilla (2017)	2	1	0	1	4

Smith CE, *et al. Tob Control* 2021; 30:e87–e95. doi: 10.1136/tobaccocontrol-2020-055874
